# Supplementary material for: Pathway-specific enzymes from bamboo and crop leaves biosynthesize anti-nociceptive C-glycosylated flavones
Source: Commun Biol. 2020 Mar 6;3:110. doi: 10.1038/s42003-020-0834-3 (PMC7060329; doi:10.1038/s42003-020-0834-3)
Supplement: Supplementary file 1 — Supplementary Infomation [file 42003_2020_834_MOESM1_ESM.pdf]

# Supplementary Methods

## Plant materials, animals, strains and chemicals

*P. meyeri* was collected from Anji County (E 119.64°, N 30.50°), Zhejiang Province, China in Oct. 2017 and March, 2018. *P. glauca*, *P. heterocycla* cv. Pubescens (Synonym: *P. edulis*), *P. prominens* and *P. bissetii* were collected from Shanghai Chenshan Botanical Gardens in April. 2018. Voucher specimens (No. Pm1710, Pm1803, Pg1804, Ph1804, Pp1804 and Pb1804) has been deposited at CAS-Key Laboratory of Synthetic Biology, Shanghai, China. Other plant materials including *O. sativa* japonica (cv. Nipponbare), *O. sativa* indica (cv. Teqing), *S. bicolor* (cv. Jingza16), *S. italic* (cv. Jinyuxiang), *B. distachyon* (diploid inbred line Bd21), *T. aestivum* (cv. Zhongyuan98-68) and *Z. mays* (cv. B73) used in this study were grown in a greenhouse at Shanghai Institute of Plant Physiology and Ecology (SIPPE), CAS. The young leaves of *Phyllostachys* bamboos collected in spring and leaves of one-week-old seedling of Gramineae crops were cut into small pieces and immediately frozen in liquid nitrogen for further use. ICR male mice were provided by the Shanghai Experimental Animal Breeding Center, CAS. Experimental procedures were conducted using groups of 6~8-week-old ICR male mice (25-30 g). The animals were randomly housed under controlled temperature (22 ± 1 °C), humidity (45-65%) and light (12 h light/12 h dark; light was switched on at 06:00 a.m.) and received food and water *ad libitum*. Groups of 10 animals were employed in each experiment. *E. coli* DH10B was used for plasmid construction and propagation. *E. coli* BL21(DE3) was used for expression of CGT genes and *de novo* production of flavone *C*-glycosides. Chemical standards phloretin (Phr), 2-hydroxylnaringenin (2OHNar), naringenin (Nar), vitexin (Vit), isovitexin (Isovit), orientin (Ori) and isoorientin (Isoori) were purchased from Dalian Meilun Biotechnology Co., Ltd (Dalian, China). Nothofagin (Nof, phloretin 3'-*C*-glucoside) and phloretin *C*-arabinoside were prepared by our laboratory and confirmed by NMR (Supplementary Fig. 13) and MS analysis (Supplementary Fig. 11). UDP-glucose

(UDP-Glc, Realtimes Biotechnology Co., Ltd., Beijing, China) and UDP-arabinose (UDP-Ara, CarboSource, U.S.A) were used as sugar donors in the enzymatic assays.

## Cloning and heterologous expression of *C*-glycosyltransferases

Genomic DNA (gDNA) of each plant species was extracted by the Plant Genomic DNA Kit (Tiangen, Beijing) according to the manufacturer's protocol. Candidate *CGT* genes (contain no introns according to the genomic analysis) were amplified directly from the gDNA by PCR using high-fidelity PrimeSTAR Max DNA polymerase (Takara, Japan) and gene-specific primers (Supplementary Table 7), except for *OsUGT708A2*, *OsUGT708A39*, *SbUGT708A38*, *ZmUGT708A6*, *TaUGT708A52*, *TaUGT708A54*, *SiUGT708A31* and *SiUGT708A33* that were synthesized and codon-optimized by GenScript Biotech (Nanjing, China). The CDS of *PhUGT708A43* (PH01001494G0270) was wrongly predicted by genome annotation[1], lacking the N-terminal. We re-defined the start codon of this gene through protein sequence alignment with other clade A1 UGT708. For the cloning of CGTs orthologous to PhUGT708A43, the same pair of primers Ph708-1-F/R was used. The CGT-coding sequences were inserted into pET28a between the *Nde*I/*Not*I sites via ClonExpress II One Step Cloning Kit (Vazyme, Nanjing, China) or plus One step PCR Cloning Kit (NovoRec, Shanghai, China). The purified plasmids were transformed into *E. coli* BL21(DE3) for the expression of target proteins. Single positive clones were inoculated into 2mL Luria-Bertani (LB) media and cultivated for 12 hours at 37 ℃, and then inoculated into 100 mL of LB medium at the ratio of 1:100 with kanamycin (50 μg/mL). When the $OD_{600}$ reached 0.5, a final concentration of 0.1 mM isopropyl β-D-1-thiogalactopyranoside (IPTG) was added for inducing expression of target CGTs. After induction for additional 20 h at 16 ℃, the cells were collected by centrifugation (6000 rpm, 5 min), The resulting suspension was lysed by using a sonication homogenizer (50 W, five cycles) in the presence of 1 mM phenylmethylsulfonyl fluoride (PMSF) protease inhibitor and 2 U DNaseI over ice for 30 min. The lysate was centrifuged (10000 rpm, 45 min) to remove insoluble cell debris.

The crude protein extracts were stored at −20 °C for subsequent purification. Ni NTA Magarose Beads (Shanghai Chuzhi Biological Technology, Shanghai, China) was used to purify the $His_6$-tagged protein. The purified UGTs were checked by SDS-PAGE (10% Tris-HCl gel) and western bolt (Supplementary Fig. 7).

## Identification of *C*-glucosylated flavones and *C*-glycosylated phloretin

In order to confirm the production of four representative *C*-glucosylated flavones, we isolated the four products (Ori, Isoori, Vit and Isovit) from 1 L fermentation of strain SCZ67. The fermentation broth was extracted by *n*-butanol (1 L for 3 times), followed by evaporation of the combined organic layers. The residues were dissolved in 20% methanol, subjected to ODS silica gel column (YMC-gel ODS-A-HG, 12 nm, S-50 μm, 250 g) and eluted with increasing gradient of methanol (from 20% to 100%) in $H_2O$. A fraction eluted with 50% methanol, which contains the *C*-glucosylated flavones and *p*-coumaric acid, was further purified repeatedly by ODS silica gel column to yield purified Ori (4.5 mg), Isoori (2.5 mg), Vit (3.2 mg) and Isovit (2.1 mg).

Nothofagin (Nof, phloretin 3'-*C*-β-D-glucoside) and putative phloretin *C*-arabinoside were prepared in 20 mL of enzymatic reactions in identical condition to the enzymatic assays. The reaction mixtures were quenched by 20 mL methanol. Denatured proteins were removed through centrifugation (5000 rpm, 15 min) and the supernatants were concentrated. The resulting residues were dissolved in 20% methanol (1 mL), filtered and further purified by semi-preparative HPLC (Dionex UltiMate 3000 Semi-Preparative HPLC Systems, Thermo Scientific, MA, U.S.A.) with a linear gradient elution of 20% to 60% (25 min) methanol in $H_2O$ (flow rate of 10 mL/min, detected at 280 nm) and SilGreen ODS column (φ 20 × 250 mm, S-5 μM) (Greenherbs Co., Ltd., Beijing, China) to yield Nof (5.2 mg) and putative phloretin *C*-arabinoside (1.0 mg).

$^1H$, $^{13}C$ and 2D NMR spectra (Supplementary Fig. 13, 17) were recorded at 25 °C on AVANCE-500 (500 MHz for $^1H$) spectrometer (Bruker BioSpin, Rheinstetten, Germany). The chemical shifts (ppm) were referenced to the solvent (DMSO-$d_6$) peaks

at $\delta_H$ = 2.50 ppm and $\delta_C$ = 39.5 ppm.

## Evaluation of cytokine production and antioxidant activity

Samples of mice paw tissue were collected for cytokine (TNF-α, IL-1β and IL-10) level determination using enzyme-linked immunosorbent assay. An hours after the injection of formalin, mice injected with formalin or saline were euthanized, and the skin tissues were removed from the treated and control paws. The samples were homogenized in 500 μL of buffer containing protease inhibitors, and TNF-α, IL-1β, IL-10 (Liuhe bio, China), GSH, MDA, VC and SOD (Nanjing Jiancheng Bioengineering Institute, China) levels were determined by an enzyme-linked immunosorbent assay (ELISA) using kits

## Supplementary Figures

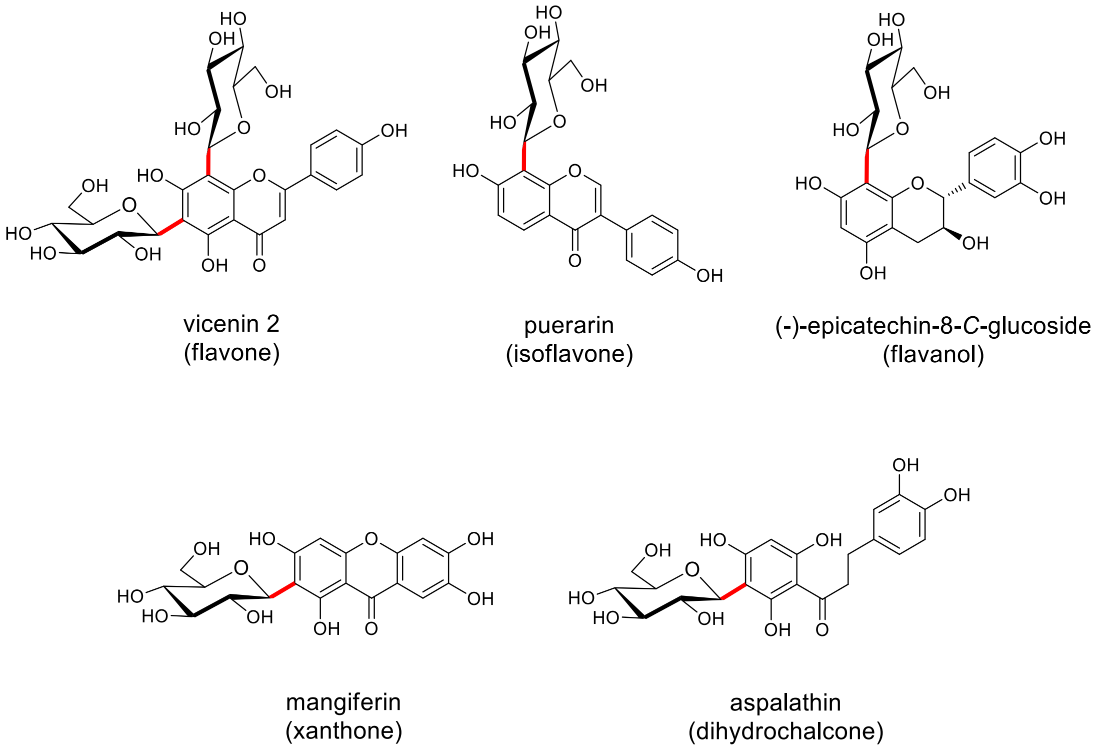


Supplementary Fig. 1 Representative structures of natural *C*-glycosides from plants.

*C*-glycosidic linkages (highlighted in red bold bonds) are found in secondary metabolites including flavone, isoflavone, flavonol, xanthone and dihydrochalcone.

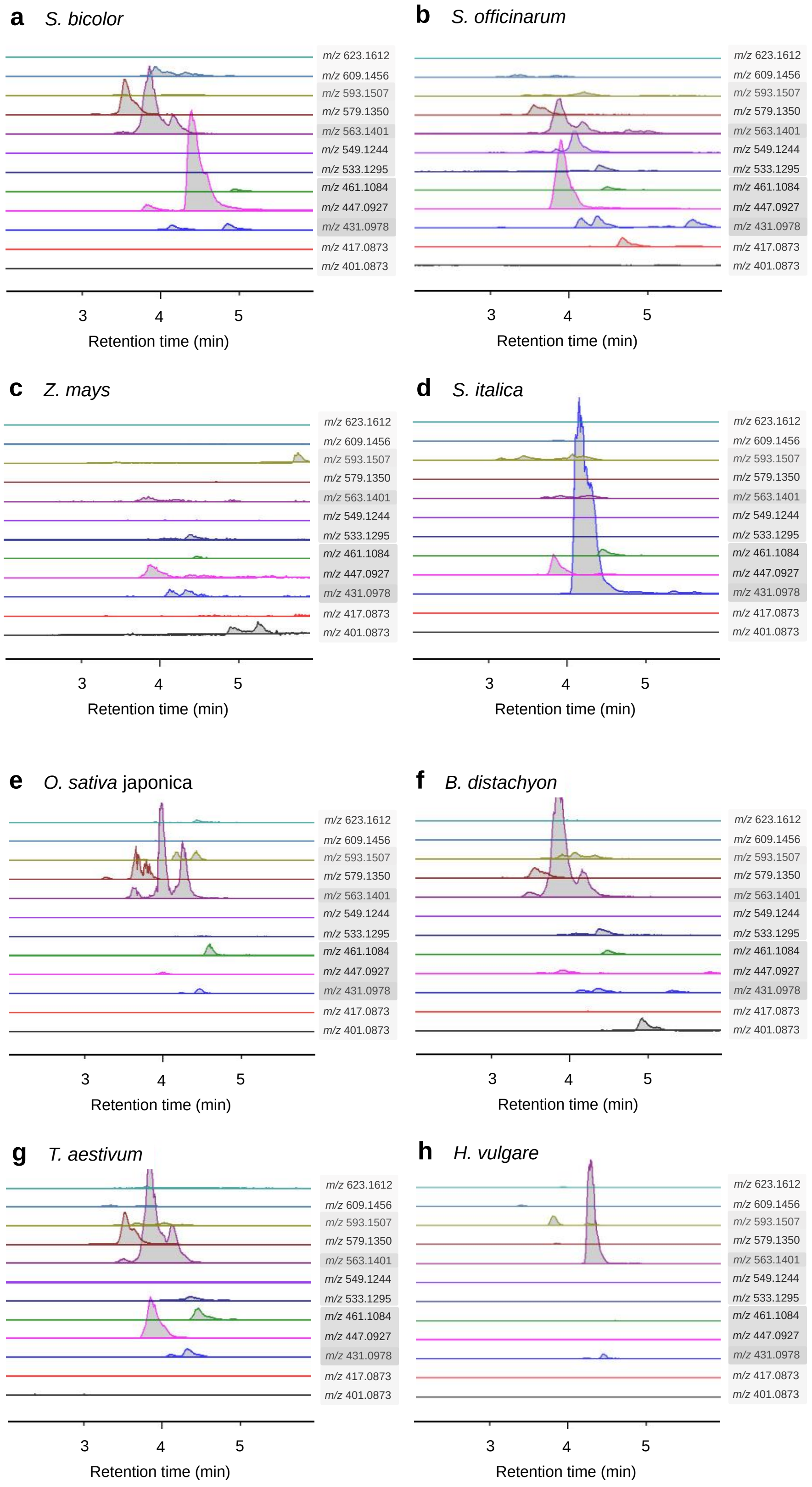
a S. bicolor
b S. officinarum
c Z. mays
d S. italica
e O. sativa japonica
f B. distachyon
g T. aestivum
h H. vulgare
m/z 623.1612
m/z 609.1456
m/z 593.1507
m/z 579.1350
m/z 563.1401
m/z 549.1244
m/z 533.1295
m/z 461.1084
m/z 447.0927
m/z 431.0978
m/z 417.0873
m/z 401.0873
3
4
5
Retention time (min)

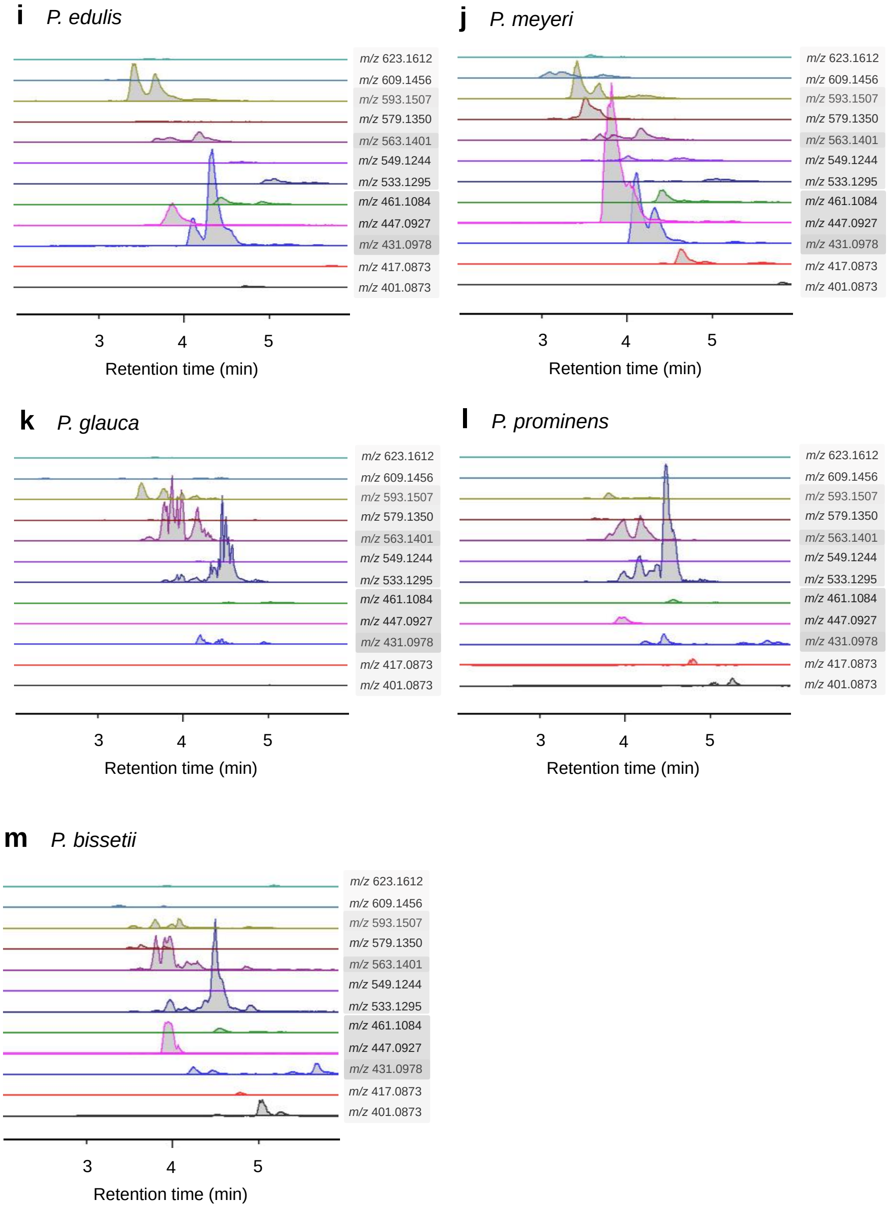


Supplementary Fig. 2 Extracted-ion chromatogram (XIC) of the *n*-butanol extracts of Gramineae plants.

The selected crops include *S. bicolor* (**a**), *S. officinarum* (**b**), *Z. mays* (**c**), *S. italic* (**d**), *O. sativa* japonica (**e**), *B. distachyon* (**f**), *T. aestivum* (**g**) and *H. vulgare* (**h**). Five *Phyllostachys* bamboos include *P. heterocycla* (**i,** = *P. edulis*, MS/MS spectra are shown in Supplementary Fig. 3), *P. meyeri* (**j**), *P. glauca* (**k**), *P. prominens* (**l**) and *P. bissetii* (**m**). Ion chromatograms corresponding to different *C*-glycosylated forms of apigenin, luteolin and chrysoeriol were extracted.

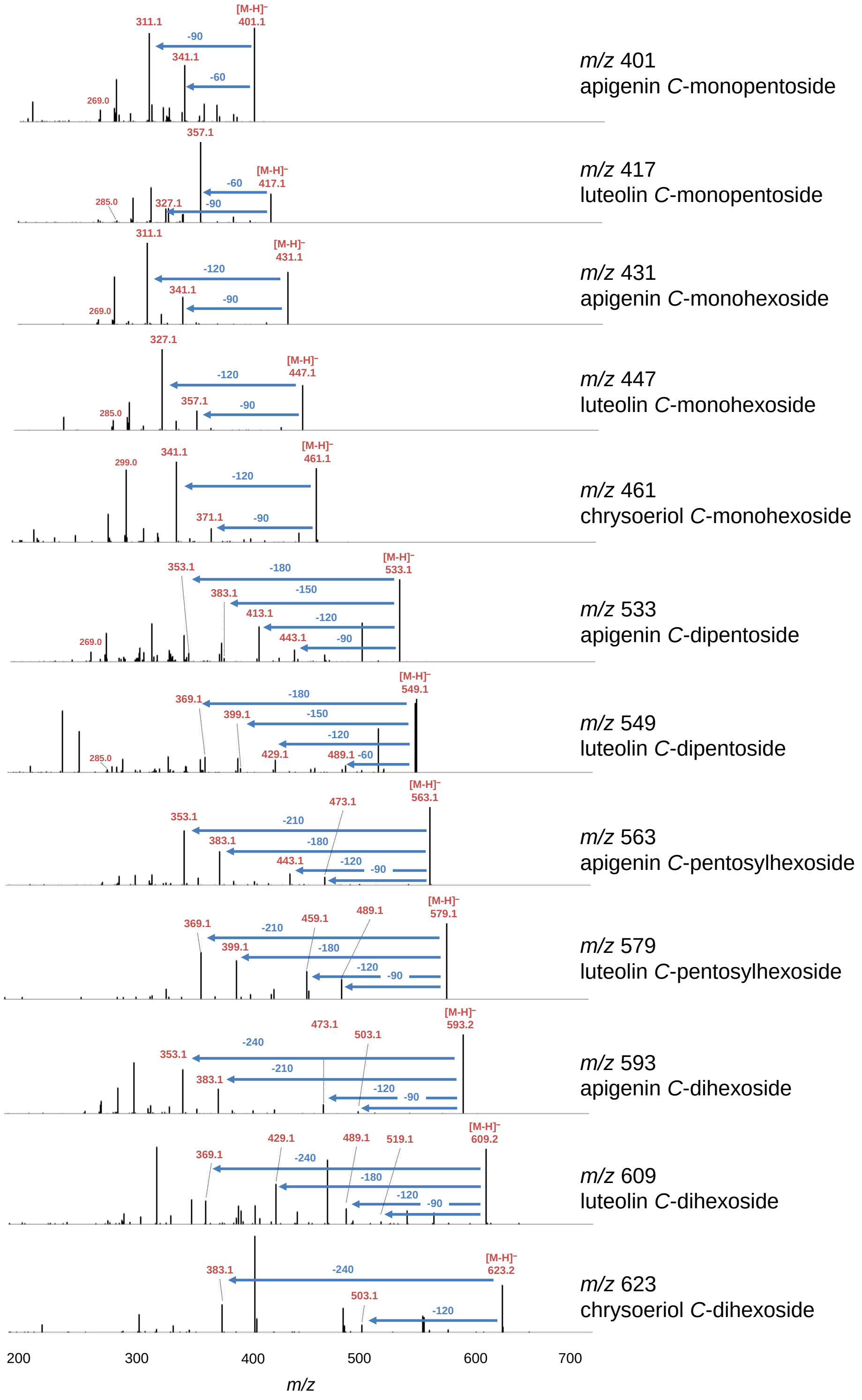


Supplementary Fig. 3 MS/MS spectra of targeted *C*-glycosides in *P. heterocycla.*

The loss of characteristic *m/z* 60, *m/z* 90 and their combination were observed in *C*-pentosides, while *C*-hexosides show $[M-H-90]^-$ and $[M-H-120]^-$ peaks as expected.

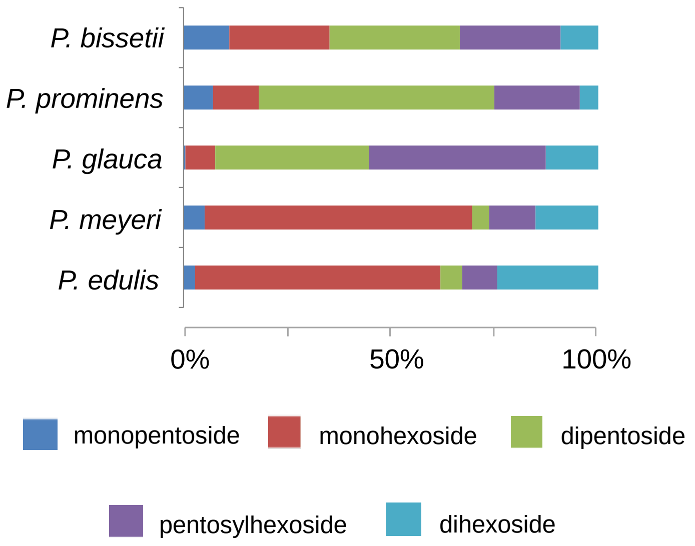


Supplementary Fig. 4 Profiling of *C*-glycosylated core structures in *Phyllostachys* bamboos.

The proportion of different glycosylated forms are displayed in different color. The major accumulated glycosides in *P. heterocycla* and *P. meyeri* are monohexoside represented by orientin and vitexin. On the other hand, *P. glauca*, *P. prominens* and *P. bissetii* basically produce more flavone dipentosides and pentosylhexosides.

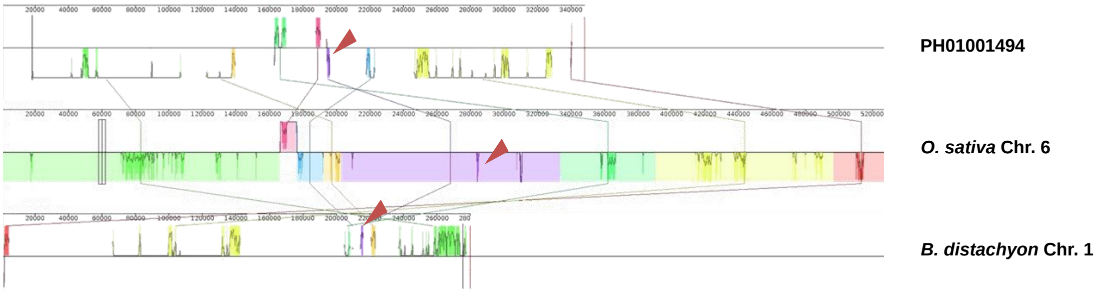


Supplementary Fig. 5 Alignment of moso bamboo scaffold PH01001494 with the chromosome 6 of *O. sativa* and the chromosome 1 of *B. distachyon.*

The location of UGT708-encoding genes is indicated in red triangles. The conserved genomic sequence was aligned by mauve [2].

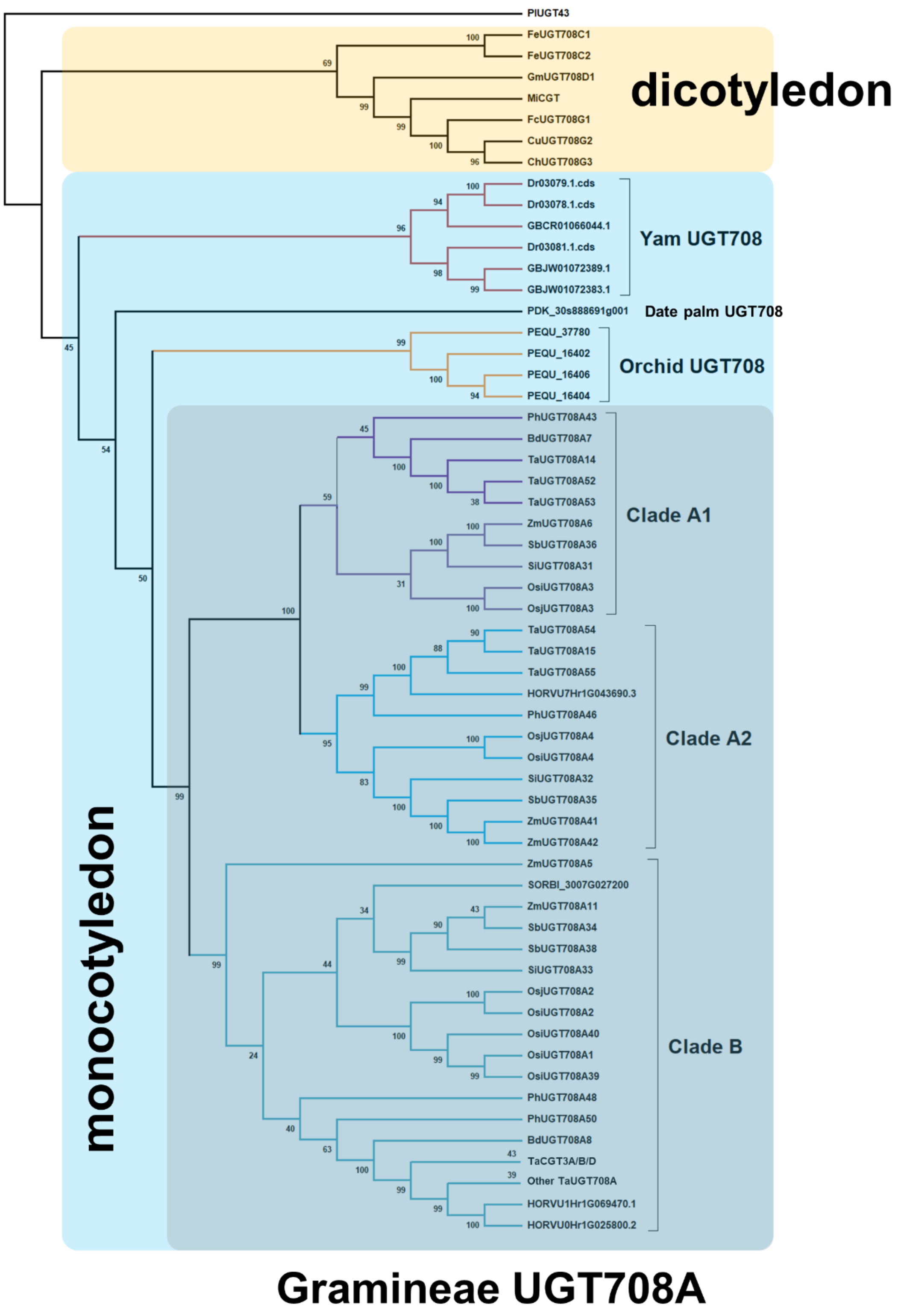


Supplementary Fig. 6 Phylogenetic tree of UGT708 family.

Bootstrap values (based on 1000 replications) are indicated at each node. PlUGT43 (KU317801.2)[3] is used as an out-group. Dicot UGT708 enzymes include MiCGT

(KT200208.1)[4], FeUGT708C1 (BAP90360.1), FeUGT708C2 (BAP90361.1)[5], GmUGT708D1 (LC003312.1)[6], FcUGT708G1 (LC131333.1), CuUGT708G2 (LC131334.1) and ChUGT708G3 (LC131335.1)[7]. Gramineae-specific *UGT708A* genes (Supplementary Table 3) are divided into Clade A1, A2 and Clade B. Information of the other monocotyledonous UGT708s from orchid, date palm and yam are listed in Supplementary Table 4.

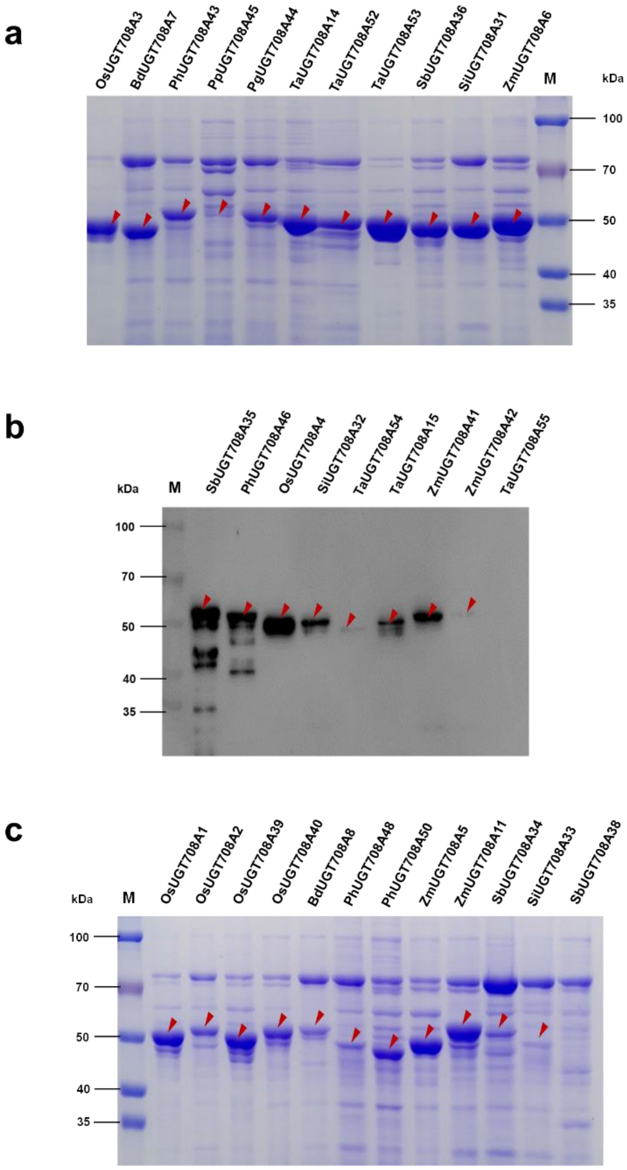


Supplementary Fig. 7 Expression of recombinant UGT708A enzymes in *E. coli*.

All the $His_6$-tagged proteins were purified by nickel magnetic beads. (**a**) SDS-PAGE indicated that Clade A1 enzymes (except for PpUGT708A45) were well expressed in *E. coli* BL21(DE3). (**b**) The expression of Clade A2 could only be detected by western blot. Although Clade A2 were not well expressed, the crude enzymes extracts were observed to be active towards Phr and 2OHNar (See Supplementary Fig. 9). (**c**) SDS-PAGE of Clade B enzymes indicated that most of them were expressed.

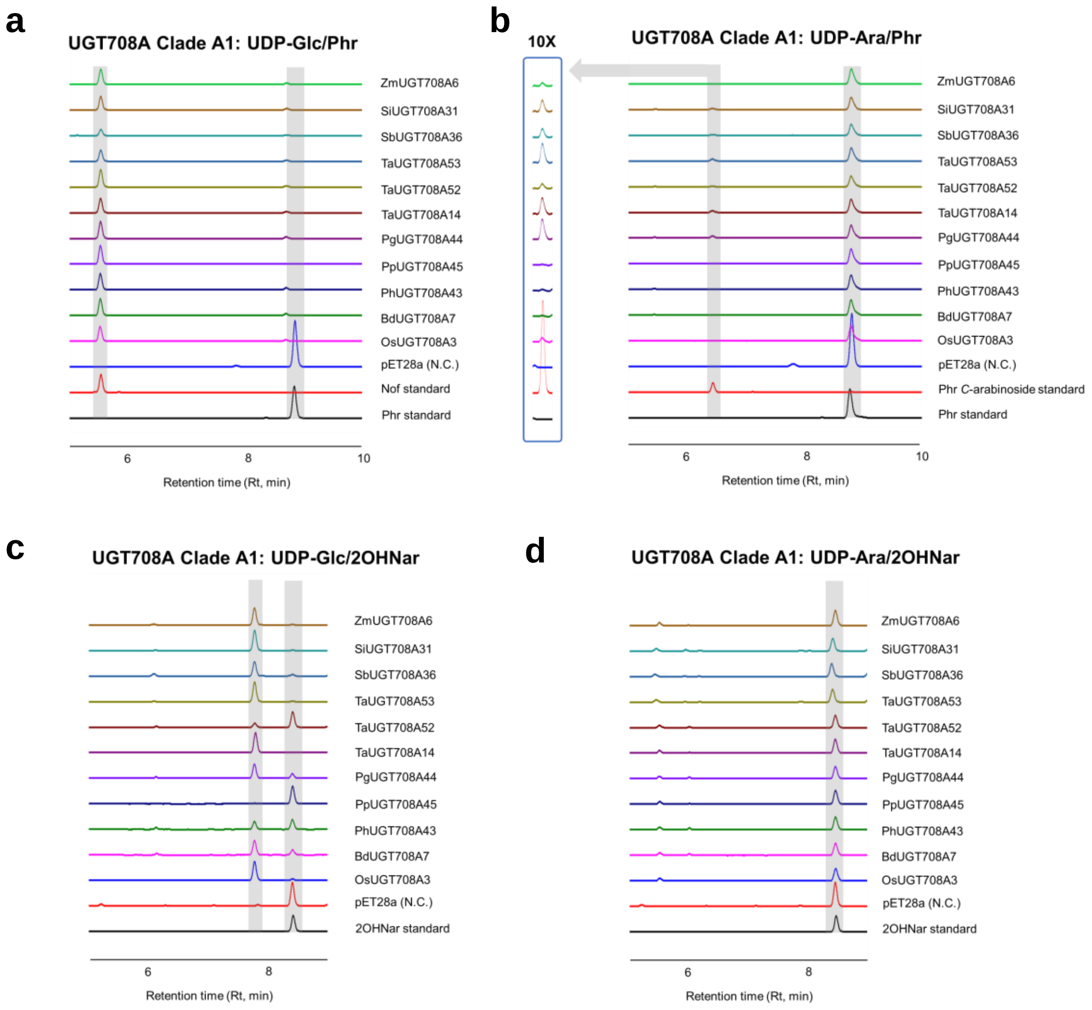


Supplementary Fig. 8 HPLC analyses of the enzymatic reaction mixtures of Clade A1 CGTs.

Eleven Clade A1 CGTs were tested towards 4 combinations of substrates: (**a**) Phr+UDP-Clc; (**b**) Phr+UDP-Ara; (**c**) 2OHNar+UDP-Clc; (**d**) 2OHNar+UDP-Ara. The Clade A1 enzymes barely generate arabinosides, but efficiently catalyze the glucosylation of Phr and 2OHNar.

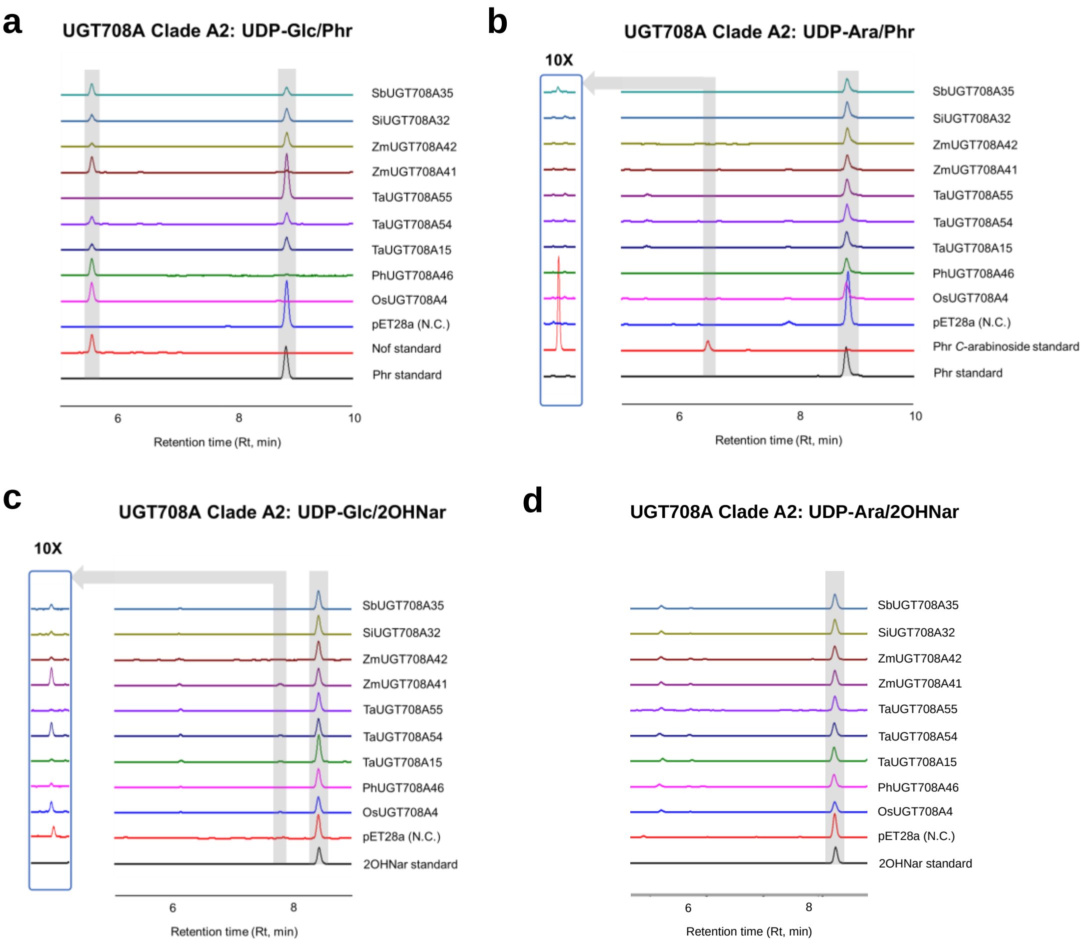


Supplementary Fig. 9 HPLC analyses of the enzymatic reaction mixtures of Clade A2 CGTs.

Clade A2 CGTs were tested towards 4 combinations of substrates: (**a**) Phr+UDP-Glc; (**b**) Phr+UDP-Ara; (**c**) 2OHNar+UDP-Glc; (**d**) 2OHNar+UDP-Ara. Clade A2 enzymes showed moderate conversion of Phr to nothofagin (Nof).

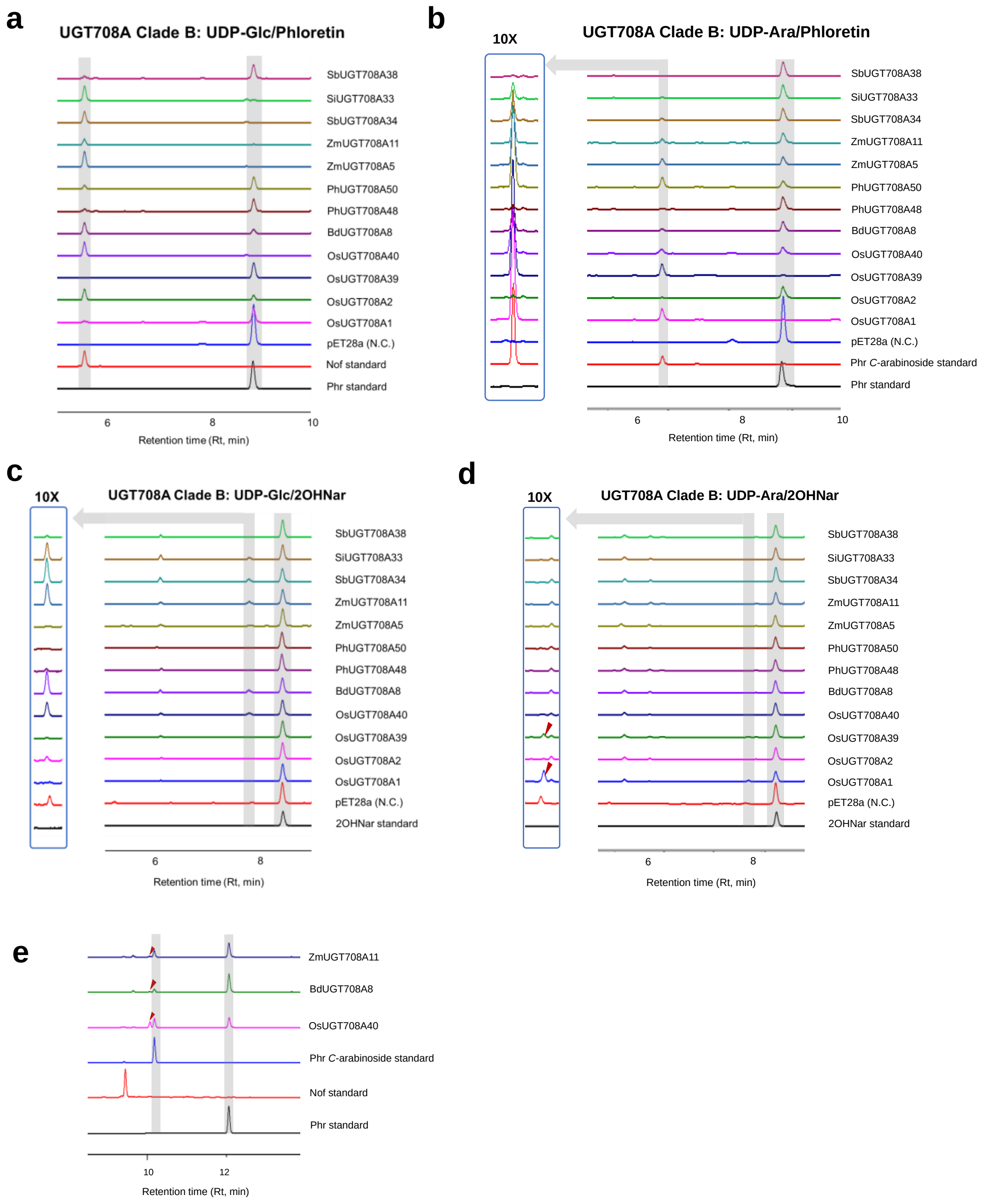


Supplementary Fig. 10 HPLC analyses of the enzymatic reaction mixtures of Clade B CGTs.

Clade B CGTs were tested towards 4 combinations of substrates: (**a**) Phr+UDP-Glc; (**b**) Phr+UDP-Ara; (**c**) 2OHNar+UDP-Glc; (**d**) 2OHNar+UDP-Ara. Clade B enzymes recognize both UDP-Glc and UDP-Ara as sugar donors. The red triangles in (**d**) indicate the proposed *C*-arabinosyl 2OHNar. (**e**) HPLC analyses revealed the presence

of proposed phloretin *C*-diarabinoside (indicated by red triangles). For LC-MS/MS spectra, see Supplementary Fig. 11c.

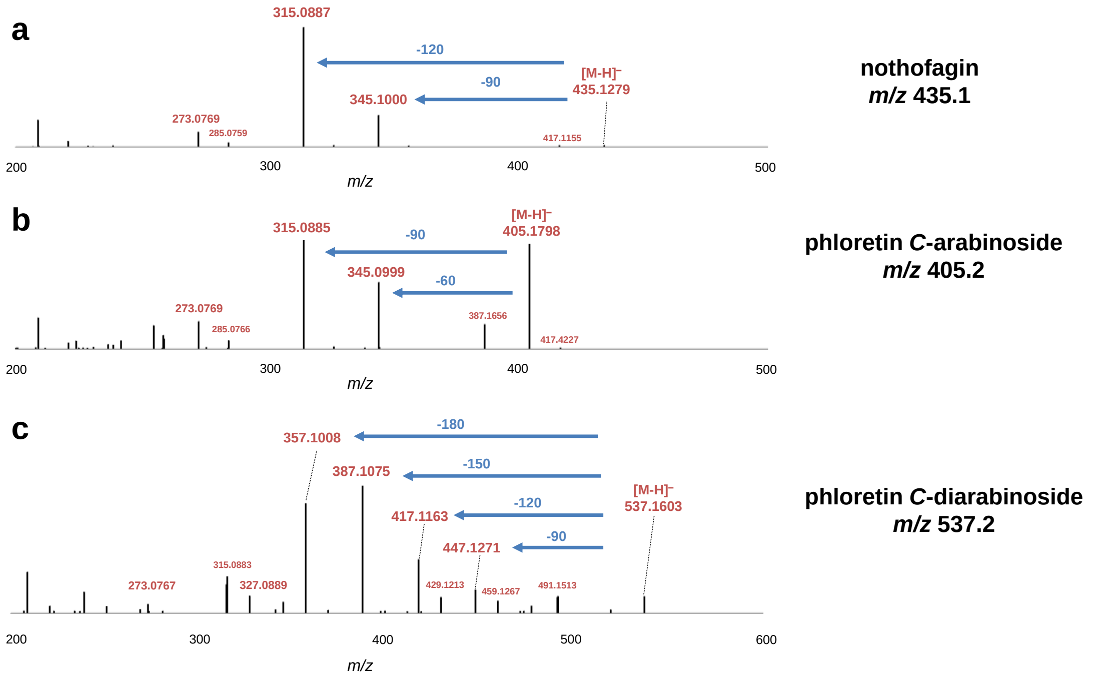


Supplementary Fig. 11 Negative mode MS/MS spectra of different glycosylated forms of phloretin (Phr).

(**a**) nothofagin, Mw = 435.1; (**b**) phloretin *C*-arabinoside, Mw = 405.2; (**c**) phloretin *C*-diarabinoside, Mw = 537.2;

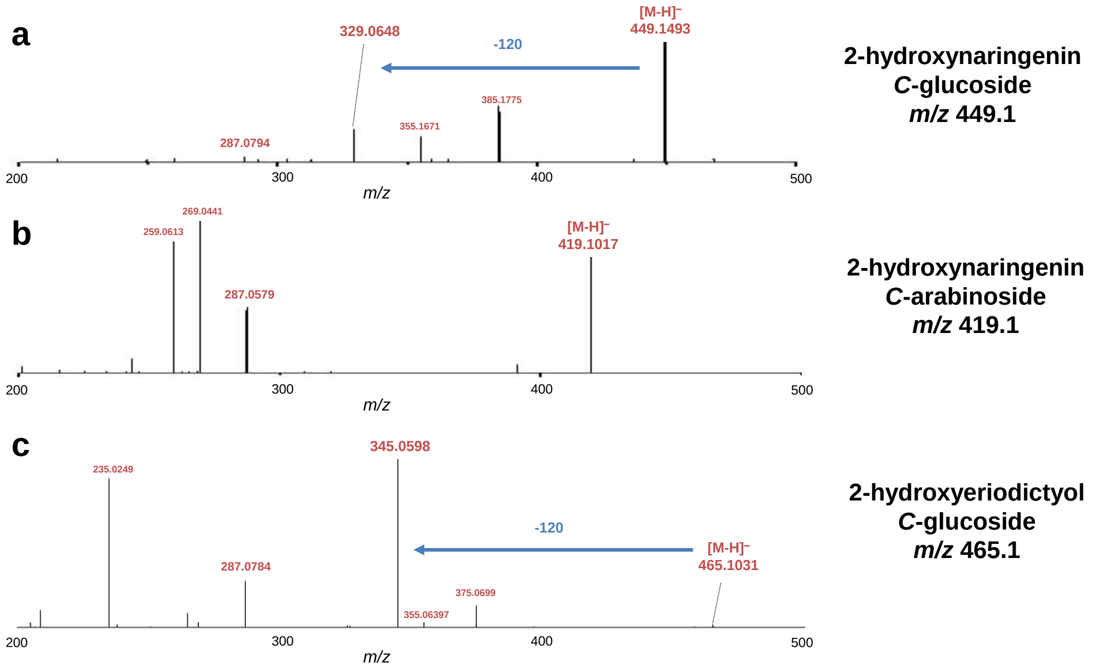


Supplementary Fig. 12 Negative mode MS/MS spectra of different glycosylated forms of 2-hydroxynaringenin (2OHNar) and 2-hydroxyeriodictyol (2OHEri).

(**a**) 2-hydroxynaringenin *C*-glucoside, Mw = 449.1; (**b**) 2-hydroxynaringenin *C*-arabinoside, Mw = 419.1, the characteristic $[M\text{-}H\text{-}120]^-$ peak was not observed, probably due to the instability of this intermediate; (**c**) 2-hydroxyeriodictyol *C*-glucoside, Mw = 465.1

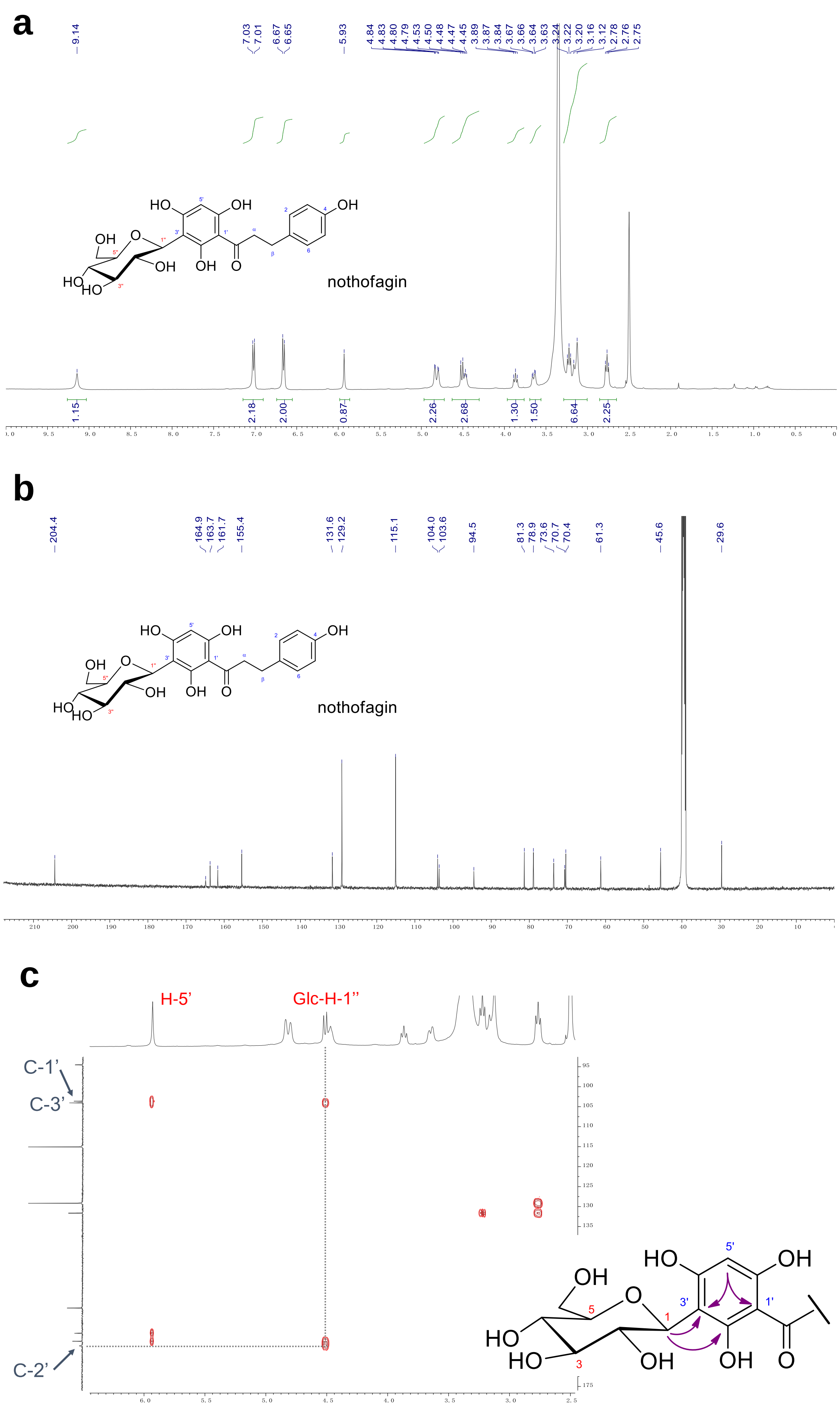
a
nothofagin
b
nothofagin
c
H-5'
Glc-H-1''
C-1'
C-3'
C-2'

**d**

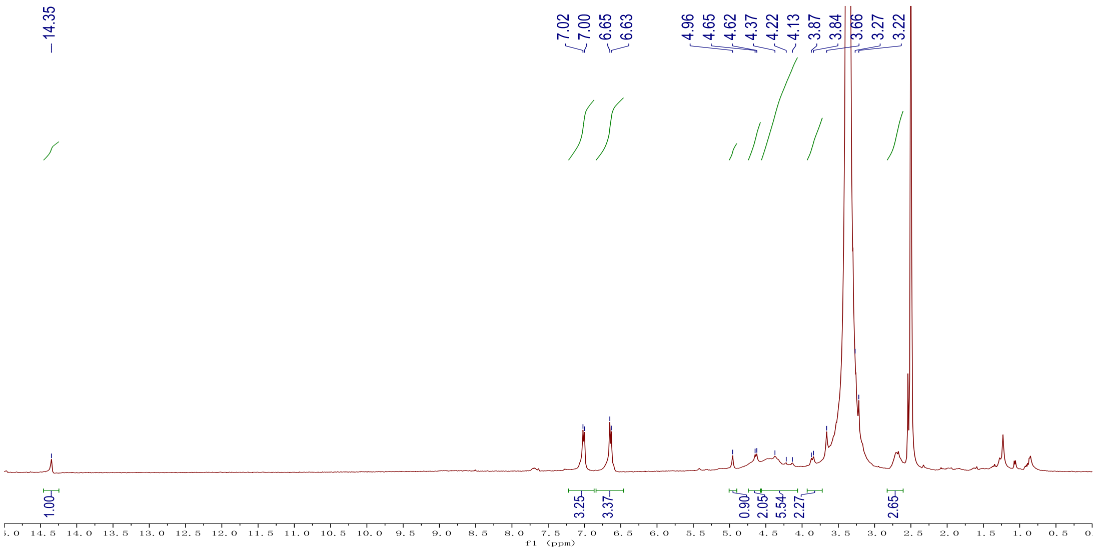


Supplementary Fig. 13 NMR spectra of nothofagin and phloretin *C*-arabinoside.

(**a**) $^{1}H$ NMR (500 MHz, DMSO-$d_6$) spectrum of nothofagin (Nof, phloretin 3'-*C*-glucoside); (**b**) $^{13}C$ NMR (125 MHz, DMSO-$d_6$) spectrum of Nof; (**c**) HMBC spectrum of Nof reveal a direct C-C linkage indicated by long-rang correlation from anomeric proton to C2' and C3'; (**d**) $^{1}H$ NMR (500 MHz, DMSO-$d_6$) spectrum of proposed phloretin *C*-arabinoside.

**a**

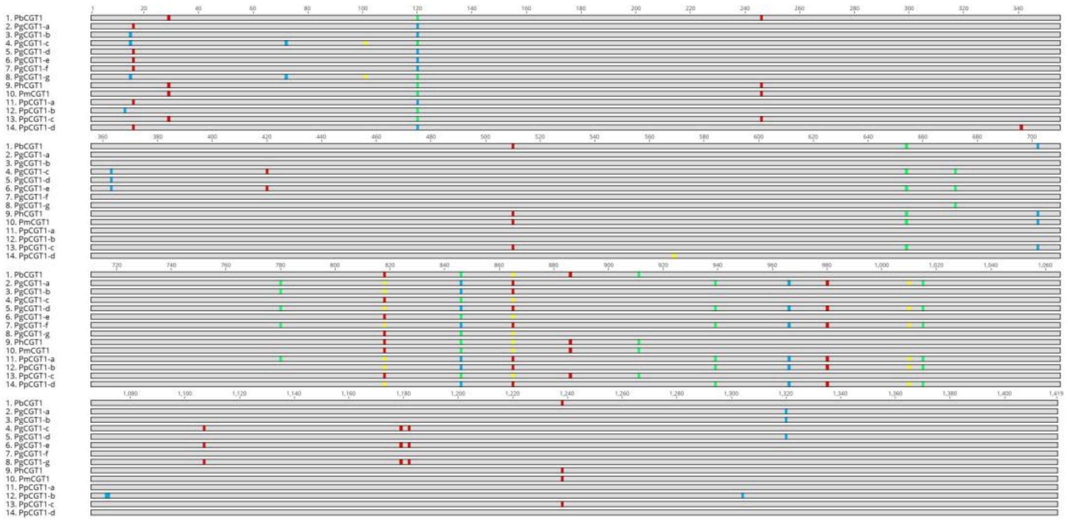

**b**

| | PbCGT1 | PgCGT1-a | PgCGT1-b | PgCGT1-c | PgCGT1-d | PgCGT1-e | PgCGT1-f | PgCGT1-g | PhCGT1 | PmCGT1 | PpCGT1-a | PpCGT1-b | PpCGT1-c | PpCGT1-d |
|---|---|---|---|---|---|---|---|---|---|---|---|---|---|---|
| PbCGT1 | | | | | | | | | | | | | | |
| PgCGT1-a | 0.985 | | | | | | | | | | | | | |
| PgCGT1-b | 0.989 | 0.995 | | | | | | | | | | | | |
| PgCGT1-c | 0.988 | 0.984 | 0.989 | | | | | | | | | | | |
| PgCGT1-d | 0.985 | 0.999 | 0.994 | 0.985 | | | | | | | | | | |
| PgCGT1-e | 0.989 | 0.988 | 0.99 | 0.996 | 0.988 | | | | | | | | | |
| PgCGT1-f | 0.986 | 0.999 | 0.994 | 0.985 | 0.998 | 0.988 | | | | | | | | |
| PgCGT1-g | 0.989 | 0.986 | 0.991 | 0.997 | 0.985 | 0.994 | 0.987 | | | | | | | |
| PhCGT1 | 1 | 0.985 | 0.989 | 0.988 | 0.985 | 0.989 | 0.986 | 0.989 | | | | | | |
| PmCGT1 | 1 | 0.985 | 0.989 | 0.988 | 0.985 | 0.989 | 0.986 | 0.989 | 1 | | | | | |
| PpCGT1-a | 0.986 | 0.999 | 0.994 | 0.985 | 0.998 | 0.988 | 1 | 0.987 | 0.986 | 0.986 | | | | |
| PpCGT1-b | 0.985 | 0.994 | 0.99 | 0.984 | 0.993 | 0.985 | 0.995 | 0.986 | 0.985 | 0.985 | 0.995 | | | |
| PpCGT1-c | 1 | 0.985 | 0.989 | 0.988 | 0.985 | 0.989 | 0.986 | 0.989 | 1 | 1 | 0.986 | 0.985 | | |
| PpCGT1-d | 0.985 | 0.997 | 0.992 | 0.984 | 0.996 | 0.988 | 0.997 | 0.986 | 0.985 | 0.985 | 0.997 | 0.994 | 0.985 | |

Supplementary Fig. 14 Comparison of *C*-glucosyltransferases from bamboos.

(**a**) Nucleotide sequence alignment of *PhUGT708A43* (*PhCGT1*) homologous genes in five *Phyllostachys* species. Seven variants were found in *P. glauca* and four variants in *P. prominens*. Sole *PhUGT708A43* (*PhCGT1*) homologies were cloned from *P. meyeri* and *P. bissetii*. (**b**) Sequence identity matrix of the cloned PhCGT1 (PhUGT708A43), PmCGT1, PgCGT1 (PgUGT708A44), PpCGT1 (PgUGT708A45) and PbCGT1 variants.

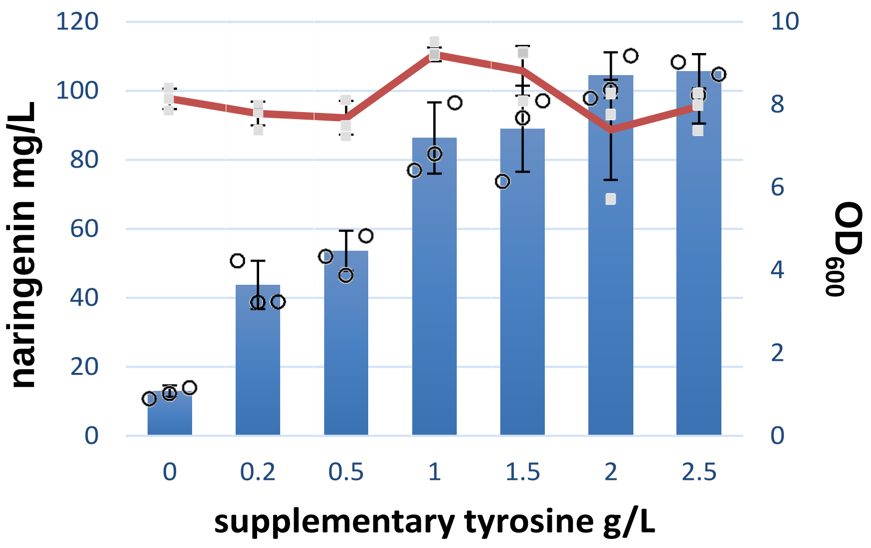


Supplementary Fig. 15 Optimization of the production of naringenin by feeding tyrosine as precursor.

The fermentation was performed for 4 days.

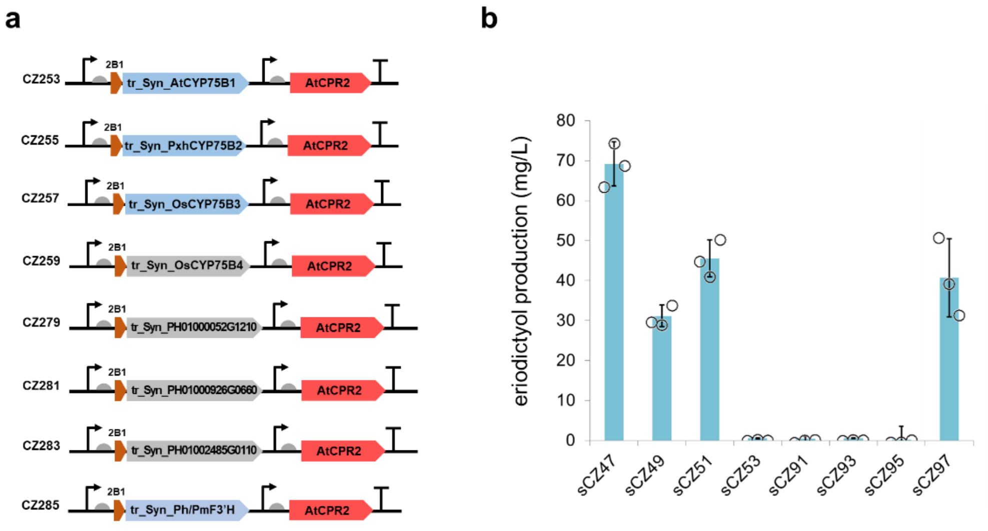


Supplementary Fig. 16 Screening of flavanone 3’-hydroxylases (F3’H).

The fermentation was performed for 4 days. (**a**) Assembled cassette of F3’H and CPR for the production of eriodictyol. (**b**) *De novo* production of eriodictyol in engineered *E. coli* strains. AtCYP75B1 gave best production of naringenin to eriodictyol (≈ 70 mg/L), representing 67% conversion rate.

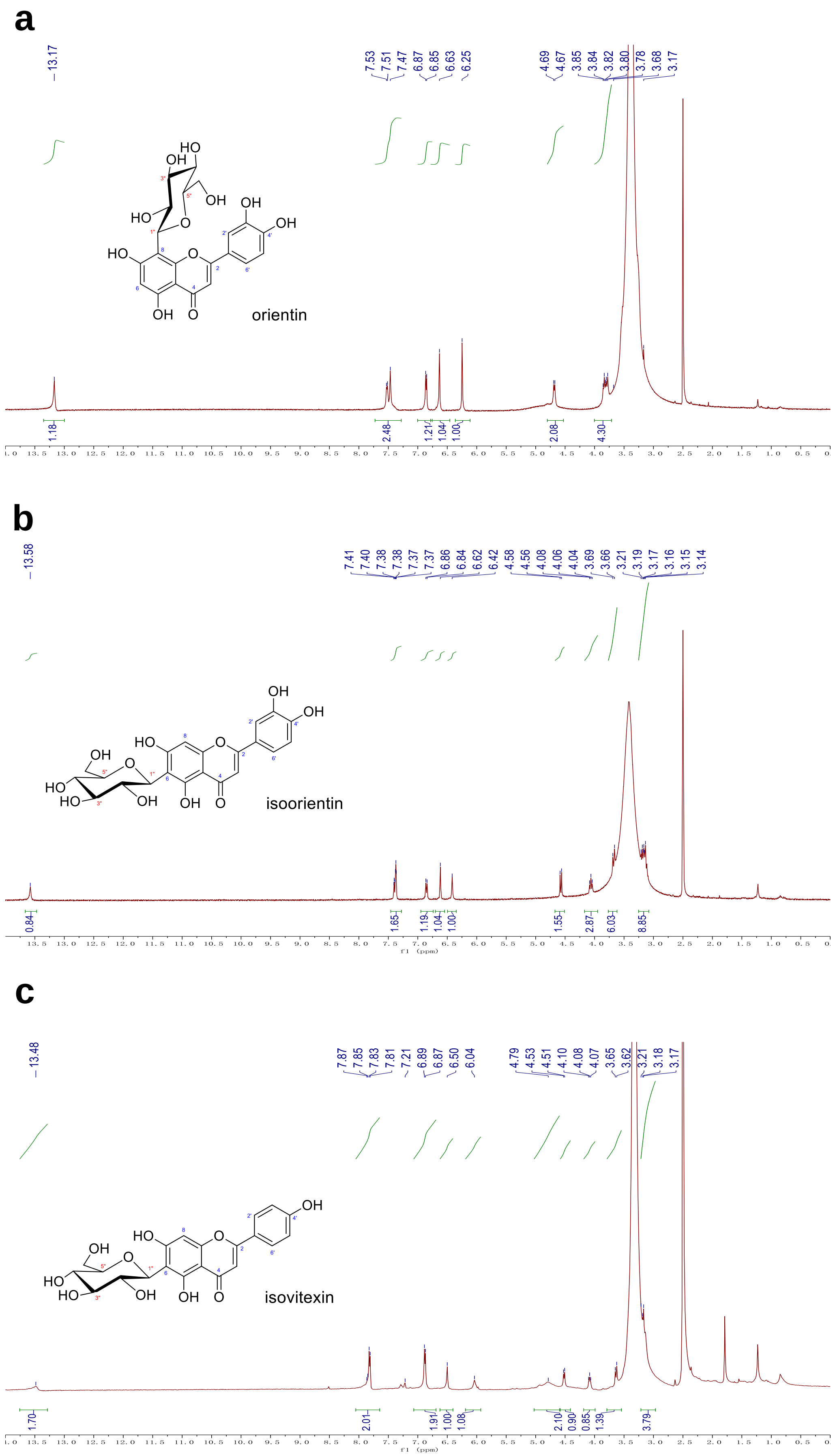
a
orientin
b
isoorientin
c
isovitexin

**d**

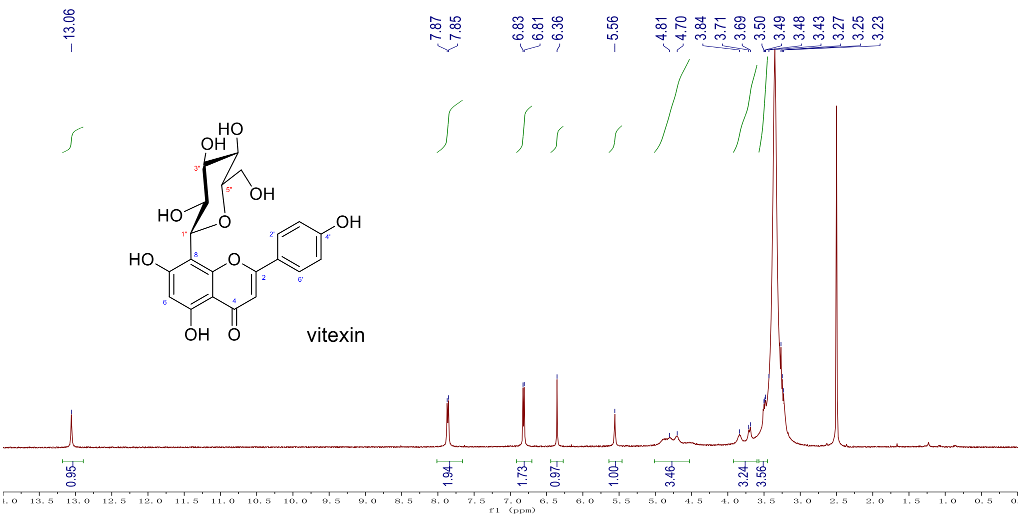


Supplementary Fig. 17 NMR analyses of flavone *C*-glucosides produced by *E. coli*.

(**a**) $^{1}$H NMR (500 MHz, DMSO-$d_6$) spectrum of the purified orientin (Ori, luteolin 8-*C*-glucoside); (**b**) $^{1}$H NMR (500 MHz, DMSO-$d_6$) spectrum of the purified isoorientin (Isoori, luteolin 6-*C*-glucoside); (**c**) $^{1}$H NMR (500 MHz, DMSO-$d_6$) spectrum of the purified isovitexin (Isovit, apigenin 6-*C*-glucoside); (**d**) $^{1}$H NMR (500 MHz, DMSO-$d_6$) spectrum of the purified vitexin (Vit, apigenin 8-*C*-glucoside).

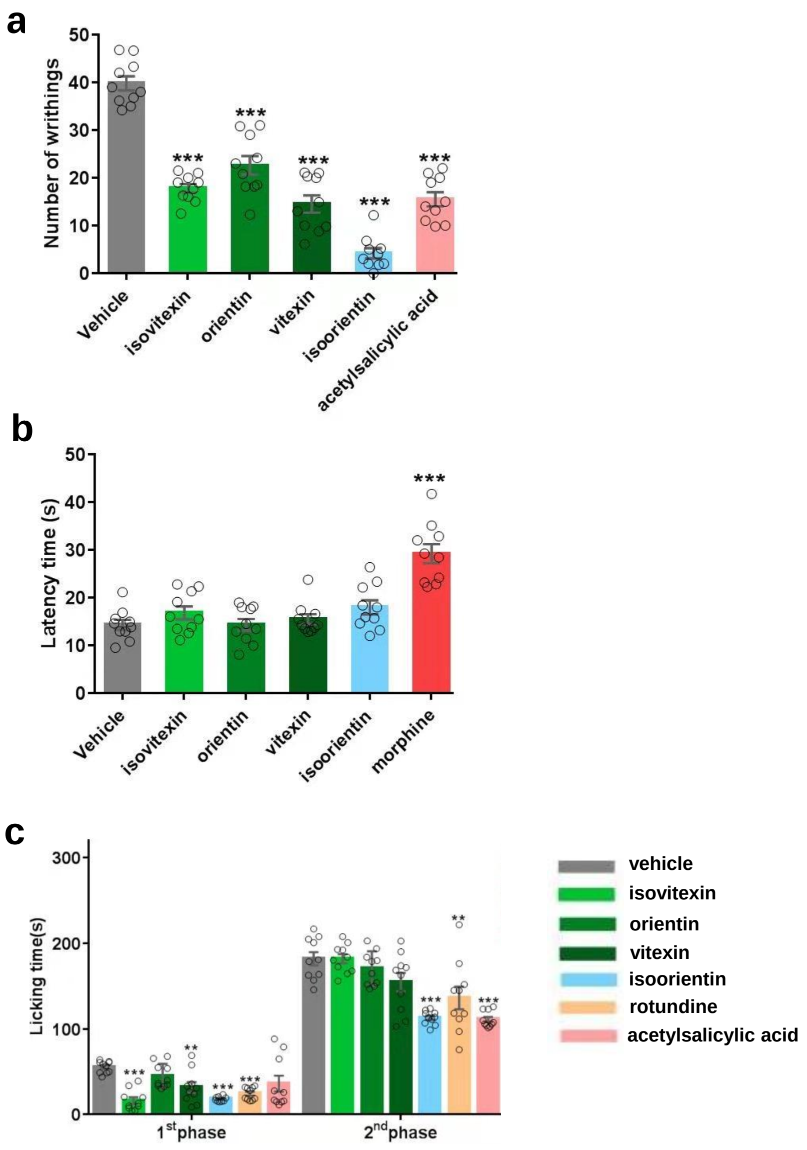


Supplementary Fig. 18 Preliminarily screening of the antinociceptive effect of *C*-glycosides.

The antinociceptive effect on (**a**) the acetic acid-induced writhing, (**b**) the latency time on the hot plate and (**c**) the first and second phase of formalin induced nociception in mice were evaluated. Isovitexin, orientin, vitexin, isoorientin, vehicle and reference drug [acetylsalicylic acid (ASA, 200 mg/kg, p.o.), morphine (MOR, 5 mg/kg, s.c.), rotundine (ROT, 20mg/kg, i.p.)] was administered to mice. Each column represents mean ± SEM (n=10). $p$<0.05 was considered as statistically significant.

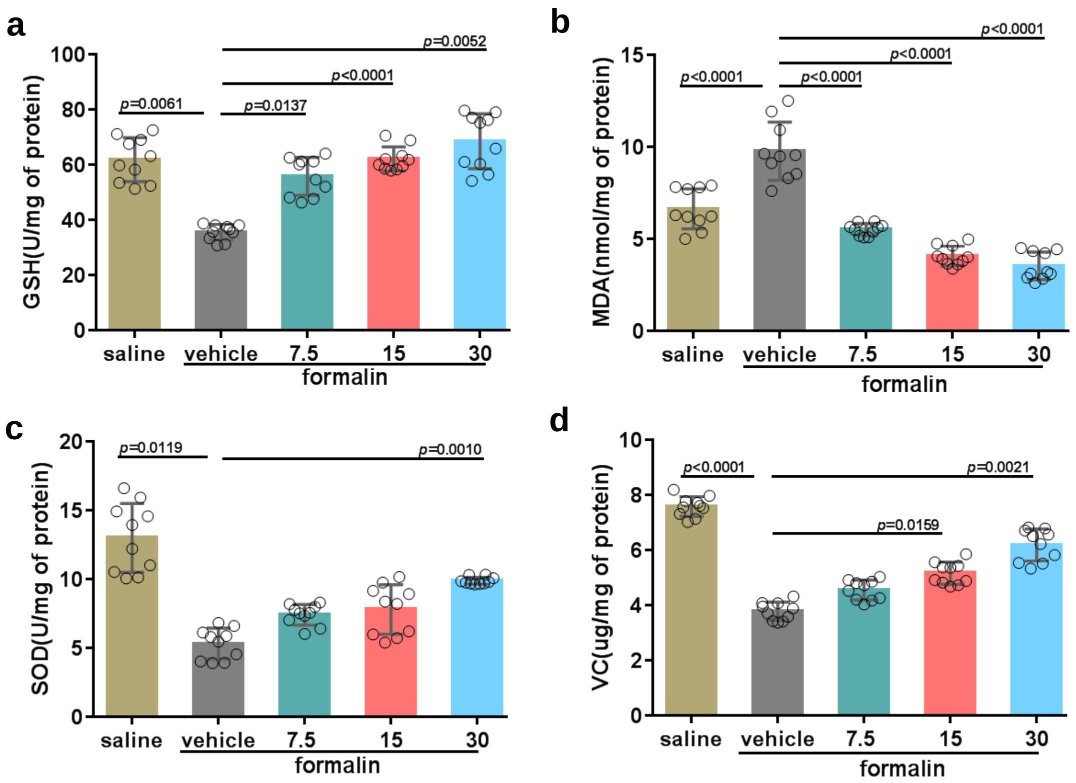


Supplementary Fig. 19 Isoorientin protects against the oxidative stress in formalin paw inflammation.

Isoorientin (7.5, 15, 30 mg/kg, i.p) increased the concentrations of GSH (**a**), SOD (**c**) and VC (**d**) in paws injected with formalin, while suppressed the formalin-stimulated MDA (**b**) in a dose-dependent manner. The concentrations of GSH, MDA, SOD and Vc were determined by ELISA. GSH, glutathione synthetase; MDA, malondialdehyde; SOD, superoxide dismutase; VC, ascorbic acid.

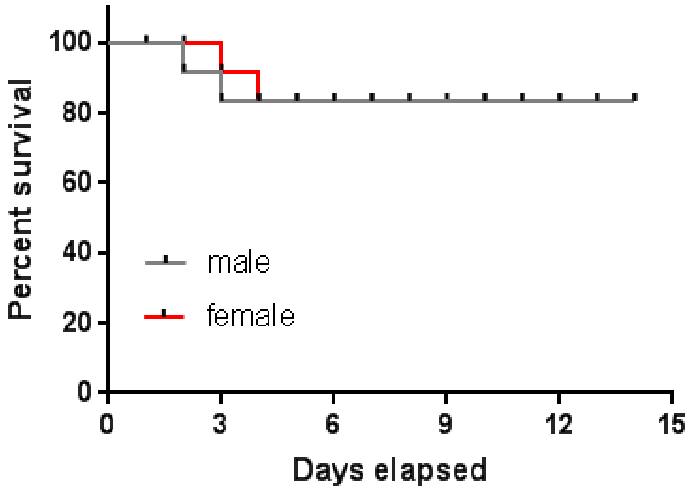


Supplementary Fig. 20 The survival curve of ICR mice oral administrated with 5000 mg/kg of isoorientin.

An oral dose of isoorientin (5000 mg/kg) was administered to a group of 14 mice (seven males and seven females) at three times with interval of 4 hours within 24 hours. Behavior parameters including convulsion, hyperactivity, sedation, grooming, loss of righting reflex, increased or decreased respiration, and food and water intake were observed and the number of the mice elapsed were counted over a period of 14 days.

## Supplementary Tables

Supplementary Table 1 Representative structures of *C*-glycosides found in monocot crops.

Sugar abbreviation: glc, glucose; ara, arabinose; rha, rhamnose.

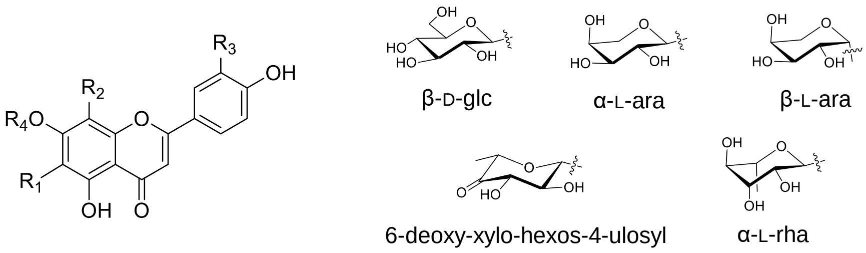


| *C*-glycoside | $R_1$ | $R_2$ | $R_3$ | $R_4$ | Origin | Ref |
|---|---|---|---|---|---|---|
| vitexin | H | β-D-glc | H | H | wheat, sugarcane | 8,9,10 |
| isovitexin | β-D-glc | H | H | H | rice, wheat | 8,11,12,13 |
| schaftoside | β-D-glc | α-L-ara | H | H | rice, wheat, sugarcane | 9,10,13,14,15 |
| isoschaftoside | α-L-ara | β-D-glc | H | H | sugarcane | 9,10 |
| neoschaftoside | β-D-glc | β-L-ara | H | H | rice | 14 |
| vicenin 2 | β-D-glc | β-D-glc | H | H | sugarcane | 16 |
| apimaysin | (2”-*O*-α-L-rha)-6-deoxy-xylo-hexos-4-ulosyl | H | H | H | maize | 17 |
| isovitexin 7-*O*-glucoside | β-D-glc | H | H | β-D-glc | barley | 18 |
| swertisin | β-D-glc | H | H | Me | rice | 15,19 |
| orientin | H | β-D-glc | OH | H | wheat, sugarcane | 8,9 |
| isoorientin | β-D-glc | H | OH | H | rice, wheat, barley | 8,12,13,18 |
| carlinoside | β-D-glc | α-L-ara | OH | H | rice, sugarcane | 14,15,16 |
| isocarlinoside | α-L-ara | β-D-glc | OH | H | sugarcane | 16 |
| neocarlinoslde | β-D-glc | β-L-ara | OH | H | rice | 14 |
| maysin | (2”-*O*-α-L-rha)-6-deoxy-xylo-hexos-4-ulosyl | H | OH | H | maize | 20 |
| derhamaysin | 6-deoxy-xylo-hexos-4-ulosyl | H | OH | H | maize | 17 |
| isoorientin 7-*O*-glucoside | β-D-glc | H | OH | β-D-glc | barley | 18 |
| lucenin 7-methyl ether | β-D-glc | β-D-glc | OH | Me | wheat | 13 |
| luteolin 8-*C*-arabinosyl-7-*O*-rhamnoside | H | α-L-ara | OH | α-L-rha | sugarcane | 16 |
| isoscoparin | β-D-glc | H | $OCH_3$ | H | rice, wheat | 8,12 |
| 3'-methoxymaysin | (2”-*O*-α-L-rha)-6-deoxy-xylo-hexos-4-ulosyl | H | $OCH_3$ | H | maize | 17 |

Supplementary Table 2 Classification of *C*-glycosylated flavone core structures (aglycones bearing only *C*-sugars).

Flavonoid *C*-glycosides in the leaves of grass family exclusively derive from apigenin, luteolin and chrysoeriol. *C*-sugars contain hexoses like glucose and galactose, and pentoses like arabinose and xylose. The *m/z* range corresponding to $[M\text{-}H]^-$ exact mass ± 3 ppm were extracted and visualized in Supplementary Fig. 2.

| Type | Aglycone | Sugar 1 | Sugar 2 | Formula | $[M\text{-}H]^-$ exact mass | Representative compound |
|---|---|---|---|---|---|---|
| | Apigenin | pentosyl | / | $C_{20}H_{18}O_9$ | 401.0873 | / |
| monopentoside | Luteolin | pentosyl | / | $C_{20}H_{18}O_{10}$ | 417.0822 | / |
| | chrysoeriol | pentosyl | / | $C_{21}H_{20}O_{10}$ | 431.0978 | / |
| | apigenin | hexosyl | / | $C_{21}H_{20}O_{10}$ | 431.0978 | vitexin |
| monohexoside | Luteolin | hexosyl | / | $C_{21}H_{20}O_{11}$ | 447.0927 | orientin |
| | chrysoeriol | hexosyl | / | $C_{22}H_{22}O_{11}$ | 461.1084 | scoparin |
| | apigenin | pentosyl | pentosyl | $C_{25}H_{26}O_{13}$ | 533.1295 | / |
| dipentoside | Luteolin | pentosyl | pentosyl | $C_{25}H_{26}O_{14}$ | 549.1244 | / |
| | chrysoeriol | pentosyl | pentosyl | $C_{26}H_{28}O_{14}$ | 563.1401 | / |
| | apigenin | hexosyl | pentosyl | $C_{26}H_{28}O_{14}$ | 563.1401 | schaftoside |
| pentosylhexoside | Luteolin | hexosyl | pentosyl | $C_{26}H_{28}O_{15}$ | 579.1350 | carlinoside |
| | chrysoeriol | hexosyl | pentosyl | $C_{27}H_{30}O_{15}$ | 593.1507 | / |
| | apigenin | hexosyl | hexosyl | $C_{27}H_{30}O_{15}$ | 593.1507 | vicenin-2 |
| dihexoside | Luteolin | hexosyl | hexosyl | $C_{27}H_{30}O_{16}$ | 609.1456 | lucenin-2 |
| | chrysoeriol | hexosyl | hexosyl | $C_{28}H_{32}O_{16}$ | 623.1612 | / |

Supplementary Table 3 Monocot UGT708A enzymes used for phylogenetic study and functional characterization.

Besides the enzymes listed in this table, several UGT708A enzymes from *H. vulgare* (*HORVU7Hr1G043690, HORVU1Hr1G069470, HORVU0Hr1G025800*), *S. bicolor* (*SORBI_3007G027200* on Chr. 7) and *T. aestivum* (UGT708 proteins on Chr.1A/B/D) were also included in Supplementary Fig. 6, although they were not cloned. TaCGT3-A/B/D were also failed to be cloned.

[a] OsCGT was reported by Brazier-Hicks, *et al.*,[21], ZmCGT was reported as a difunctional *C*- and *O*-glucosyltransferase by Ferreyra, *et al.*,[22]

[b] Accession numbers provided here are Uniprot IDs, except for those of bamboo CGTs which are deposited in Genbank.

[c] These proteins are highly similar to OsUGT708A2, OsUGT708A3 and OsUGT708A4 in japonica rice (>99.9% identity).

[d] The cloned *PmCGT1* and *PbCGT1* are completely identical to *PhCGT1(PhUGT708A43)*.

[e] Multiple variants were obtained. For details, see Supplementary Fig. 14.

| Name | UGT Name | Gene name | Accession[b] | Origin | Chr. | Location | Length (bp) | Protein (AA) | clade |
|---|---|---|---|---|---|---|---|---|---|
| OsCGT[a] | OsUGT708A3 | Os06g0288300 | Q5VMI0 | *O. sativa* japonica | 6 | 10479285-10480865 | 1416 | 471 | A1 |
| OsCGT2 | OsUGT708A4 | Os06g0289200 | Q5VMG8 | *O. sativa* japonica | 6 | 10553309-10555041 | 1458 | 485 | A2 |
| OsCGT3 | OsUGT708A2 | Os06g0289900 | Q5VME5 | *O. sativa* japonica | 6 | 10588158-10589935 | 1446 | 481 | B |
| OsCGT4 | OsUGT708A1 | OsI_22597 | A2YBW7 | *O. sativa* indica | 6 | 11449286-11450716 | 1431 | 476 | B |
| | OsUGT708A2i[c] | OsI_22594 | A2YBW4 | *O. sativa* indica | 6 | 11400834-11402279 | 1446 | 481 | B |
| | OsUGT708A3i[c] | OsI_22585 | B8B0N6 | *O. sativa* indica | 6 | 11342794-11344209 | 1416 | 471 | A1 |

*continued*

| | | | | | | | | | |
|---|---|---|---|---|---|---|---|---|---|
| | OsUGT708A4i[c] | OsI_22591 | A2YBW1 | *O. sativa* indica | 6 | 11381748-11383205 | 1458 | 485 | A1 |
| OsCGT5 | OsUGT708A39 | OsI_22598 | A2YBW8 | *O. sativa* indica | 6 | 11452927-11454357 | 1431 | 476 | B |
| OsCGT6 | OsUGT708A40 | OsI_22600 | A2YBX1 | *O. sativa* indica | 6 | 11478827-11480266 | 1440 | 479 | B |
| SbCGT1 | SbUGT708A36 | SORBI_3010G120600 | C5Z8Y8 | *S. bicolor* | 10 | 13902238-13904019 | 1431 | 476 | A1 |
| SbCGT2 | SbUGT708A35 | SORBI_3010G120400 | C5Z8Y5 | *S. bicolor* | 10 | 13829175-13831497 | 1449 | 482 | A2 |
| SbCGT3 | SbUGT708A34 | SORBI_3010G120200 | C5Z8Y2 | *S. bicolor* | 10 | 13705963-13707854 | 1464 | 487 | B |
| SbCGT4 | SbUGT708A38 | SORBI_3010G012400 | A0A194YGR9 | *S. bicolor* | 10 | 1061213-1062715 | 1380 | 459 | B |
| TaCGT1-A | TaUGT708A14 | / | A0A3B6RGT4 | *T. aestivum* | 7A | 182,384,453-182,386,078 | 1455 | 484 | A1 |
| TaCGT1-B | TaUGT708A52 | / | A0A3B6SEV6 | *T. aestivum* | 7B | 144,483,417-144,485,194 | 1419 | 472 | A1 |
| TaCGT1-D | TaUGT708A53 | / | A0A3B6TPG6 | *T. aestivum* | 7D | 178,291,737-178,293,161 | 1425 | 474 | A1 |
| TaCGT2-A | TaUGT708A15 | / | A0A3B6RGR9 | *T. aestivum* | 7A | 182,561,465-182,562,874 | 1410 | 469 | A2 |
| TaCGT2-B | TaUGT708A54 | / | A0A3B6SB95 | *T. aestivum* | 7B | 144,517,418-144,519,262 | 1410 | 469 | A2 |
| TaCGT2-D | TaUGT708A55 | / | A0A3B6TNL8 | *T. aestivum* | 7D | 178,474,995-178,476,419 | 1425 | 474 | A2 |
| TaCGT3-A | / | / | A0A3B6RC34 | *T. aestivum* | 7A | 156,549,057-156,550,919 | 1431 | 476 | B |
| TaCGT3-B | / | / | A0A3B6SBI7 | *T. aestivum* | 7B | 116,753,251-116,755,445 | 1425 | 474 | B |
| TaCGT3-D | / | / | A0A1D6CY58 | *T. aestivum* | 7D | 154,934,104-154,935,896 | 1428 | 475 | B |
| ZmCGT[a] | ZmUGT708A6 | Zm00001d037382 | A0A1Q1B4E0 | *Z. mays* | 6 | 123791605-123793032 | 1428 | 475 | A1 |
| ZmCGT2 | ZmUGT708A5 | Zm00001d037383 | B6SWX3 | *Z. mays* | 6 | 123804940-123806574 | 1449 | 482 | A/B |
| ZmCGT3 | ZmUGT708A11 | Zm00001d037384 | A0A1D6LXE6 | *Z. mays* | 6 | 123931487-123933195 | 1452 | 483 | B |
| ZmCGT4 | ZmUGT708A41 | Zm00001d045788 | B4FAN3 | *Z. mays* | 9 | 39266100-39267810 | 1440 | 479 | A2 |
| ZmCGT5 | ZmUGT708A42 | Zm00001d045862 | B4FM13 | *Z. mays* | 9 | 44291551-44293313 | 1455 | 484 | A2 |

*Continued*

| | | | | | | | | | |
|---|---|---|---|---|---|---|---|---|---|
| BdCGT1 | BdUGT708A7 | BRADI_1g43410v3 | I1GYZ6 | *B. distachyon* | 1 | 41292902-41296125 | 1419 | 472 | A1 |
| BdCGT2 | BdUGT708A8 | BRADI_1g44630v3 | I1GZD7 | *B. distachyon* | 1 | 42932272-42934452 | 1464 | 487 | B |
| SiCGT1 | SiUGT708A31 | SETIT_006342mg | K3XWN4 | *S. italica* | 4 | 13,240,590-13,242,244 | 1428 | 475 | A1 |
| SiCGT2 | SiUGT708A32 | SETIT_006299mg | K3XWJ1 | *S. italica* | 4 | 13,242,609-13,244,801 | 1485 | 494 | A2 |
| SiCGT3 | SiUGT708A33 | SETIT_007944mg | K3Y181 | *S. italica* | 4 | 13,258,567-13,259,995 | 1404 | 467 | B |
| PhCGT1 | PhUGT708A43 | PH01001494G0270 | MK616588[b] | *P. heterocycla* | / | | 1419 | 472 | A1 |
| PhCGT2 | PhUGT708A46 | PH01000603G0510 | MK616589[b] | *P. heterocycla* | / | | 1416 | 471 | A2 |
| PhCGT3 | PhUGT708A48 | PH01005199G0080 | MK616590[b] | *P. heterocycla* | / | | 1422 | 473 | B |
| PhCGT4 | PhUGT708A50 | PH01000051G0480 | MK616591[b] | *P. heterocycla* | / | | 1425 | 474 | B |
| PmCGT1[d] | / | | / | *P. meyeri* | / | | 1419 | 472 | A1 |
| PgCGT1[e] | PgUGT708A44 | | MK616592[b] | *P.glauca* | / | | 1419 | 472 | A1 |
| PpCGT1[e] | PpUGT708A45 | | MK616593[b] | *P. prominens* | / | | 1419 | 472 | A1 |
| PbCGT1[d] | / | | / | *P. bissetii* | / | | 1419 | 472 | A1 |

Supplementary Table 4 Non-grass family monocotyledonous UGT708 in banana, date palm, yam and orchids.

No *UGT708* genes was found in banana (*Musa acuminata*). Date palm, yam and orchid possess 2~6 UGT708-encoding genes, though most of these genes are fragments.

| Origin | Common Name | Order | Family | Sequence Assembly | UGT708 | ID | Len (AA) | Type | Ref |
|---|---|---|---|---|---|---|---|---|---|
| *Musa acuminata* | banana | Zingiberales | Musaceae | ASM31385v1 (Genome) | 0 | / | / | / | 23 |
| *Phoenix dactylifera* | date palm | Arecales | Arecaceae | PDK30 (v3) (Genome) | 2 | PDK_30s866991g001 | 265 | fragment | 24 |
| | | | | | | PDK_30s888691g001 | 309 | fragment | |
| *Dioscorea composita* | Barbasco | Dioscoreales | Dioscoreaceae | GBJW01.1 (Transcriptome) | 6 | GBJW01072389.1 | 444 | full-length | / |
| | | | | | | GBJW01072383.1 | 444 | full-length | |
| | | | | | | GBJW01072385.1 | 211 | fragment | |
| | | | | | | GBJW01072384.1 | 198 | fragment | |
| | | | | | | GBJW01019112.1 | 168 | fragment | |
| | | | | | | GBJW01019111.1 | 118 | fragment | |
| *Dioscorea rotundata* | white Guinea yam | Dioscoreales | Dioscoreaceae | TDr96_F1_Pseudo_Chromosome_v1.0 (Genome) | 5 | Dr03079.1.cds | 468 | full-length | 25 |
| | | | | | | Dr03081.1.cds | 460 | full-length | |
| | | | | | | Dr03078.1.cds | 359 | fragment | |
| | | | | | | Dr03080.1.cds | 277 | fragment | |
| | | | | | | Dr03077.1.cds | 776 | fragment | |
| *Dioscorea zingiberensis* | peltate yam | Dioscoreales | Dioscoreaceae | GBCR01.1 (Transcriptome) | 2 | GBCR01066044.1 | 363 | fragment | 26 |
| | | | | | | GBCR01038049.1 | 162 | fragment | |
| *Phalaenopsis equestris* | / | Asparagales | Orchidaceae | OrchidBase 3.0 (Genome) | 5 | PEQU_16406 | 427 | fragment | 27 |
| | | | | | | PEQU_16402 | 386 | fragment | |
| | | | | | | PEQU_16404 | 370 | fragment | |
| | | | | | | PEQU_37780 | 324 | fragment | |
| | | | | | | PEQU_40915 | 68 | fragment | |

Supplementary Table 5 $K_m$ values of recombinant CGTs toward 2-hydroxynaringenin and phloretin.

Data are presented as means ± S.D. from triplicate measurements (n = 3).

[a] $K_m$ values were measured with UDP-glucose (400 μM) as sugar donor.

[b] Previously reported data[21].

[c] cannot be determined because no detectable conversion was observed.

| $K_m$ (μM)[a] | UDP-Glc (as sugar donor) | |
|---|---|---|
| | phloretin | 2-hydroxynaringenin |
| OsUGT708A3 | 4.78[b] | 2.5[b] |
| BdUGT708A7 | 2.72 ± 1.39 | 24.27 ± 10.37 |
| PhUGT708A43 | 15.83 ± 6.98 | 1.75 ± 0.66 |
| PpUGT708A45 | 12.73 ± 4.69 | /[c] |
| PgUGT708A44 | 12.87 ± 4.46 | 2.75 ± 0.75 |
| TaUGT708A14 | 4.57 ± 2.19 | 25.82 ± 6.62 |
| TaUGT708A52 | 7.71 ± 3.42 | /[c] |
| TaUGT708A53 | 19.01 ± 4.07 | 19.40 ± 7.55 |
| SbUGT708A36 | 4.22 ± 2.48 | 28.01 ± 7.85 |
| SiUGT708A31 | 5.32 ± 0.80 | 19.29 ± 5.42 |
| ZmUGT708A6 | 11.47 ± 2.48 | 48.31 ± 18.26 |
| OsUGT708A4 | 3.47 ± 1.73 | /[c] |
| PhUGT708A46 | 76.35 ± 41.16 | /[c] |
| TaUGT708A15 | 26.54 ± 3.33 | /[c] |
| TaUGT708A54 | 44.12± 8.37 | /[c] |
| TaUGT708A55 | /[c] | /[c] |
| ZmUGT708A41 | 39.13 ± 12.29 | /[c] |
| ZmUGT708A42 | 15.73 ± 6.71 | /[c] |
| SiUGT708A32 | 22.00 ± 9.24 | /[c] |
| SbUGT708A35 | 27.05 ± 11.59 | /[c] |
| OsUGT708A1 | /[c] | /[c] |
| OsUGT708A2 | 51.06 ± 30.64 | /[c] |
| OsUGT708A39 | /[c] | /[c] |
| OsUGT708A40 | 116.8 ± 33.7 | /[c] |
| BdUGT708A8 | 17.97 ± 3.94 | /[c] |
| PhUGT708A48 | /[c] | /[c] |
| PhUGT708A50 | /[c] | /[c] |
| ZmUGT708A5 | 36.92 ± 7.87 | /[c] |
| ZmUGT708A11 | 35.66 ± 8.29 | /[c] |
| SbUGT708A34 | 19.15 ± 4.92 | /[c] |
| SiUGT708A33 | 21.06 ± 8.50 | /[c] |
| SbUGT708A38 | /[c] | /[c] |

## Supplementary Table 6 Plasmids and strains used in this study.

| Plasmids | | Description | Usage |
|---|---|---|---|
| pET28a | | $T_7$ promoter, pBR322 ori, $Kan^R$ | CGT expression |
| pCDFDuet-1 | | double $T_7$ promoters, CloDF13 ori, $Spd^R$ | naringenin precursor module-harboring |
| pETDuet-1 | | double $T_7$ promoters, pBR322 ori, $Amp^R$ | flavonoid P450 module-harboring |
| pYH55 | pCDF-$T_7$-4CL-$T_7$-PAL-$T_7$-CHS-$T_7$-CHI | pCDFDuet-1 carrying PAL, 4CL, CHS and CHI | naringenin production[28] |
| pCZ86 | pET28a-$T_7$-PhCGT1 | pET28a carrying PhCGT1 | *C*-glycosylation |
| pCZ201 | pETDuet-$T_7$-2B1-tr29_Syn_ZmCYP93G5-$T_7$-AtCPR2 | pETDuet-1 carrying ZmF2H and CPR | flavonoid F2H screening |
| pCZ203 | pETDuet-$T_7$-2B1-tr26_Syn_OsCYP93G2-$T_7$-AtCPR2 | pETDuet-1 carrying OsF2H and CPR | flavonoid F2H screening |
| pCZ229 | pETDuet-$T_7$-2B1-tr37_Syn_SbCYP93G3-$T_7$-AtCPR2 | pETDuet-1 carrying SbF2H and CPR | flavonoid F2H screening |
| pCZ277 | pETDuet-$T_7$-2B1-tr24_Syn_Ph/PmCYP93G-$T_7$-AtCPR2 | pETDuet-1 carrying Ph/PmF2H and CPR | flavonoid F2H screening |
| pCZ253 | pETDuet-$T_7$-2B1-tr20_Syn_AtCYP75B1-$T_7$-AtCPR2 | pETDuet-1 carrying AtF3'H and CPR | flavonoid F3'H screening |
| pCZ255 | pETDuet-$T_7$-2B1-tr24_Syn_PxhCYP75B2-$T_7$-AtCPR2 | pETDuet-1 carrying PxhF3'H and CPR | flavonoid F3'H screening |
| pCZ257 | pETDuet-$T_7$-2B1-tr24_Syn_OsCYP75B3-$T_7$-AtCPR2 | pETDuet-1 carrying OsF3'H[29] and CPR | flavonoid F3'H screening |
| pCZ259 | pETDuet-$T_7$-2B1-tr28_Syn_OsCYP75B4-$T_7$-AtCPR2 | pETDuet-1 carrying OsF3'5'H[30] and CPR | flavonoid F3'H screening |
| pCZ279 | pETDuet-$T_7$-2B1-tr24_Syn_PH01000052G1210-$T_7$-AtCPR2 | pETDuet-1 carrying PH01000052G1210 and CPR | flavonoid F3'H screening |
| pCZ281 | pETDuet-$T_7$-2B1-tr26_Syn_PH01000926G0660-$T_7$-AtCPR2 | pETDuet-1 carrying PH01000926G0660 and CPR | flavonoid F3'H screening |
| pCZ283 | pETDuet-$T_7$-2B1-tr21_Syn_PH01002485G0110-$T_7$-AtCPR2 | pETDuet-1 carrying PH01002485G0110 and CPR | flavonoid F3'H screening |
| pCZ285 | pETDuet-$T_7$-2B1-tr23_Syn_Ph/PmF3'H-$T_7$-AtCPR2 | pETDuet-1 carrying Ph/PmF3'H and CPR | flavonoid F3'H screening |
| pCZ261 | pETDuet-$T_7$-2B1-tr29_Syn_ZmCYP93G5-$T_7$-2B1-tr20_Syn_AtCYP75B1-$T_7$-AtCPR2 | pETDuet-1 carrying ZmF2H, AtF3'H and CPR | *C*-glycoside production |
| pCZ265 | pETDuet-$T_7$-2B1-tr29_Syn_ZmCYP93G5-$T_7$-2B1-tr24_Syn_OsCYP75B3-$T_7$-AtCPR2 | pETDuet-1 carrying ZmF2H, OsF3'H and CPR | *C*-glycoside production |

| | | | |
|---|---|---|---|
| pCZ287 | pETDuet-$T_7$-2B1-tr24_Syn_Ph/PmCYP93G - $T_7$-2B1-tr20_Syn_AtCYP75B1-$T_7$-AtCPR2 | pETDuet-1 carrying Ph/PmF2H, AtF3'H and CPR | *C*-glycoside production |
| pCZ291 | pETDuet-$T_7$-2B1-tr24_Syn_Ph/PmCYP93G - $T_7$-2B1-tr24_Syn_OsCYP75B3-$T_7$-AtCPR2 | pETDuet-1 carrying Ph/PmF2H, OsF3'H and CPR | *C*-glycoside production |

| Strains: *E. coli* BL21(DE3) | | Resistance | Usage |
|---|---|---|---|
| sCZ2 | BL21(DE3) carrying pYH55, pCZ201 and pCZ86 | $Spd^R$, $Amp^R$, $Kan^R$ | vitexin/isovitexin production from tyrosine |
| sCZ4 | BL21(DE3) carrying pYH55, pCZ203 and pCZ86 | $Spd^R$, $Amp^R$, $Kan^R$ | vitexin/isovitexin production from tyrosine |
| sCZ9 | BL21(DE3) carrying pYH55 | $Spd^R$ | control strain |
| sCZ29 | BL21(DE3) carrying pYH55, pCZ229 and pCZ86 | $Spd^R$, $Amp^R$, $Kan^R$ | vitexin/isovitexin production from tyrosine |
| sCZ47 | BL21(DE3) carrying pYH55, pCZ253 | $Spd^R$, $Amp^R$ | eriodictyol production from tyrosine |
| sCZ49 | BL21(DE3) carrying pYH55, pCZ255 | $Spd^R$, $Amp^R$ | eriodictyol production from tyrosine |
| sCZ51 | BL21(DE3) carrying pYH55, pCZ257 | $Spd^R$, $Amp^R$ | eriodictyol production from tyrosine |
| sCZ53 | BL21(DE3) carrying pYH55, pCZ259 | $Spd^R$, $Amp^R$ | eriodictyol production from tyrosine |
| sCZ63 | BL21(DE3) carrying pYH55, pCZ261 and pCZ86 | $Spd^R$, $Amp^R$, $Kan^R$ | orientin/isoorientin production from tyrosine |
| sCZ67 | BL21(DE3) carrying pYH55, pCZ265 and pCZ86 | $Spd^R$, $Amp^R$, $Kan^R$ | orientin/isoorientin production from tyrosine |
| sCZ89 | BL21(DE3) carrying pYH55, pCZ277 and pCZ86 | $Spd^R$, $Amp^R$, $Kan^R$ | vitexin/isovitexin production from tyrosine |
| sCZ91 | BL21(DE3) carrying pYH55, pCZ279 | $Spd^R$, $Amp^R$ | eriodictyol production from tyrosine |
| sCZ93 | BL21(DE3) carrying pYH55, pCZ281 | $Spd^R$, $Amp^R$ | eriodictyol production from tyrosine |
| sCZ95 | BL21(DE3) carrying pYH55, pCZ283 | $Spd^R$, $Amp^R$ | eriodictyol production from tyrosine |
| sCZ97 | BL21(DE3) carrying pYH55, pCZ285 | $Spd^R$, $Amp^R$ | eriodictyol production from tyrosine |
| sCZ99 | BL21(DE3) carrying pYH55, pCZ287 and pCZ86 | $Spd^R$, $Amp^R$, $Kan^R$ | orientin/isoorientin production from tyrosine |
| sCZ103 | BL21(DE3) carrying pYH55, pCZ291 and pCZ86 | $Spd^R$, $Amp^R$, $Kan^R$ | orientin/isoorientin production from tyrosine |
| sSYW30 | BL21(DE3) carrying pCZ277 and pCZ86 | $Amp^R$, $Kan^R$ | vitexin/isovitexin production from naringenin |

ori: origin of replication; $T_7$: $T_7$ promoter; $Kan^R$: kanamycin resistant; $Spd^R$: spectinomycin resistant; $Amp^R$: ampicillin resistant; tr_: truncated; Syn_: synthesized, codon-optimized for *E. coli*.

## Supplementary Table 7 Primers used in this study.

| *UGT construct* | Primers | Sequence (5'→3') |
|---|---|---|
| *PhUGT708A43* | Ph708-1-F | TGCCGCGCGGCAGCCATATGATGGGCCACCTGGTGC |
| | Ph708-1-R | TGGTGCTCGAGTGCGGCCGCCTAGTCCAACACTGCAAGATCCC |
| *PhUGT708A46* | Ph708-2-F | TGCCGCGCGGCAGCCATATGATGGCCTCGCGGGC |
| | Ph708-2-R | TGGTGCTCGAGTGCGGCCGCTCACGTACTGCACCGTCGC |
| *PhUGT708A48* | Ph708-3-F | TGCCGCGCGGCAGCCATATGATGGCTCCGCCGGC |
| | Ph708-3-R | TGGTGCTCGAGTGCGGCCGCTTAATTATTCTTGAGCTTGGCAACAAACTGC |
| *PhUGT708A50* | Ph708-4-F | TGCCGCGCGGCAGCCATATGATGGCCCCACCTGAAATGC |
| | Ph708-4-R | TGGTGCTCGAGTGCGGCCGCTTAATTAGCCTTGAGCTTGGCAACAA |
| *OsUGT708A3* | Os708A3-F | TGCCGCGCGGCAGCCATATGATGCCGAGCTCTGGCGAC |
| | Os708A3-R | TGGTGCTCGAGTGCGGCCGCTCAATTAGTGCGACATGTTCCCCC |
| *OsUGT708A4* | Os708A4-F | TGCCGCGCGGCAGCCATATGATGTGTTCGGCGGCAACAC |
| | Os708A4-R | TGGTGCTCGAGTGCGGCCGCTCACATTCCGTTCGTGATGACAAGC |
| *OsUGT708A1* | Os708A1-F | TGCCGCGCGGCAGCCATATGATGGCACCGCCAACGG |
| | Os708A1-R | TGGTGCTCGAGTGCGGCCGCTTAAGTGGCCTTGAGCTTTGCAACA |
| *OsUGT708A40* | Os27-F | TGCCGCGCGGCAGCCATATGATGGCACCGCCAACGG |
| | Os27-R | TGGTGCTCGAGTGCGGCCGCTTAAGCAGCCTTGAGCTTTGCAACA |
| *ZmUGT708A5* | Zm708A5-F | TGCCGCGCGGCAGCCATATGATGGCTCCGCCGCC |
| | Zm708A5-R | TGGTGCTCGAGTGCGGCCGCTCAAGCTCCTCCCTTAAGCTTGGC |
| *ZmUGT708A11* | Zm708UGTS1-F | TGCCGCGCGGCAGCCATATGATGGCCCCGCCGG |
| | Zm708UGTS1-R | TGGTGCTCGAGTGCGGCCGCTCAAGACGCCACGGTTGCT |
| *ZmUGT708A41* | Zm708UGTS2-F | TGCCGCGCGGCAGCCATATGATGTCCTCGCCGGCAC |
| | Zm708UGTS2-R | TGGTGCTCGAGTGCGGCCGCTCATGCGGTGCAACGACG |
| *ZmUGT708A42* | Zm708UGTS3-F | TGCCGCGCGGCAGCCATATGATGTCCTCGCCGGCAC |
| | Zm708UGTS3-R | TGGTGCTCGAGTGCGGCCGCTTATATAACTTCACGCGGTGCAATGC |
| *BdUGT708A7* | Bd708A7-F | TGCCGCGCGGCAGCCATATGATGCCGACCTCCGGCG |
| | Bd708A7-R | TGGTGCTCGAGTGCGGCCGCTTACTCACGCACTCTTCCATCCCG |
| *BdUGT708A8* | Bd708A8-F | TGCCGCGCGGCAGCCATATGATGGCTGATCTGGCGGCG |
| | Bd708A8-R | TGGTGCTCGAGTGCGGCCGCTTAGGTAGTACTAGGCTTGAGCTTGGCG |
| *SbUGT708A36* | Sb2-F | TGCCGCGCGGCAGCCATATGATGGCCCCATCGGCGAT |
| | Sb2-R | TGGTGCTCGAGTGCGGCCGCCTACCACCCCGCGTCGC |
| *SbUGT708A35* | Sb708UGT-4 F | TGCCGCGCGGCAGCCATATGATGTCCTCGCCGGCATTATCA |
| | Sb708UGT-4 R | TGGTGCTCGAGTGCGGCCGCTCACGCCGTGCAACGAC |
| *SbUGT708A34* | Sb708UGT-1 F | TGCCGCGCGGCAGCCATATGATGGCACCGCCGGC |
| | Sb708UGT-1 R | TGGTGCTCGAGTGCGGCCGCTCAAGGGCTGCAGTGCG |
| *TaUGT708A14* | Ta1A F | TGCCGCGCGGCAGCCATATGATGCCGACCTCCGGCG |
| | Ta1A R | TGGTGCTCGAGTGCGGCCGCTTATTCCGTGTTCACGCTTCTTCTAAATGTAGC |
| *TaUGT708A53* | Ta1A F | TGCCGCGCGGCAGCCATATGATGCCGACCTCCGGCG |
| | Ta1D R | TGGTGCTCGAGTGCGGCCGCTTACTTGCTGACGCTTAGATCCCGG |

| | | |
|---|---|---|
| *TaUGT708A15* | Ta2A-F | TGCCGCGCGGCAGCCATATGATGGCCTCCAGCTCGAGAGAC |
| | Ta2A-R | TGGTGCTCGAGTGCGGCCGCTTACTGTAAGGCACTACCATGACAGCAG |
| *TaUGT708A55* | Ta2D-F | TGCCGCGCGGCAGCCATATGATGGCCTCCAGCTCCAGAG |
| | Ta2D-R | TGGTGCTCGAGTGCGGCCGCTCACGCCTCTGACGGCG |
| *SiUGT708A32* | Si2-F | TGCCGCGCGGCAGCCATATGATGTCCTCACCGCCACCG |
| | Si2-R | TGGTGCTCGAGTGCGGCCGCTCATCCTCGCGCTGTCCACG |

| P450 module | Primers | Sequence (5'→3') |
|---|---|---|
| *pCZ201* | CZ201-F | AGCTCCCACCAGGACCTAGCATGAGCACCTGGTCCAACCG |
| | CZ201-R | GAGCTCGAATTCGGATCCACTAGTTTAGGTCGCCGCACGCGCC |
| *pCZ203* | CZ203-F | CTCCCACCAGGACCTAGCATGCGTAGCGCGGGTAGCCG |
| | CZ203-R | TCGAATTCGGATCCACTAGTTTAGCTATAAAAGCTCGGCAGCG |
| *pCZ229* | CZ229-F | AGCTCCCACCAGGACCTAGCATGATCATTCGTTGGCGTTGGAACA |
| | CZ229-R | GAGCTCGAATTCGGATCCACTAGTTTAGGTCGCTTTACCGGTCGC |
| *pCZ253* | CZ253-F | AAGCTCCCACCAGGACCTAGCATGAGCCACCGTCGTAACCGTA |
| | CZ253-R | GAGCTCGAATTCGGATCCACTAGTTTAACCGCTGCCCAGACCAT |
| *pCZ255* | CZ255-F | AGCTCCCACCAGGACCTAGCATGCGTAAACGTTATCCGCTGCCG |
| | CZ255-R | GAGCTCGAATTCGGATCCACTAGTTTAGCCGATATACGCCTGCGC |
| *pCZ257* | CZ257-F | AGCTCCCACCAGGACCTAGCATGCTGCGTGGTGGCAG |
| | CZ257-R | GAGCTCGAATTCGGATCCACTAGTTTAAACGCCATACGCGCTCG |
| *pCZ259* | CZ259-F | AGCTCCCACCAGGACCTAGCATGAGCCGTGCGGGTAAAG |
| | CZ259-R | GAGCTCGAATTCGGATCCACTAGTTTACGCGATGTTGTACGCGC |
| *pCZ261* | CZ261-F | CGACCTAAACTAGTGGATCCTAATACGACTCACTATAGGGGAATTGTGAG |
| | CZ261-R | CTTAAGCATTATGCGGCCGCTTAACCGCTGCCCAGACCAT |
| *pCZ265* | CZ265-F | CGACCTAAACTAGTGGATCCTAATACGACTCACTATAGGGGAATTGTGAG |
| | CZ265-R | CTTAAGCATTATGCGGCCGCTTAAACGCCATACGCGCTCGG |
| *pCZ277* | CZ277-F | AGCTCCCACCAGGACCTAGCATGGGCAGCGCGGG |
| | CZ277-R | TCGAATTCGGATCCACTAGTTTACGCCAGCGCCG |
| *pCZ287* | CZ287-F | CGGCGCGCCTGCAGGTCGACTAATACGACTCACTATAGGGGAATTGTGAGC |
| | CZ287-R | CTTAAGCATTATGCGGCCGCTTAACCGCTGCCCAGACCAT |
| *pCZ291* | CZ291-F | CGGCGCGCCTGCAGGTCGACTAATACGACTCACTATAGGGGAATTGTGAGC |
| | CZ291-R | CTTAAGCATTATGCGGCCGCTTAAACGCCATACGCGCTCGG |

## Supplementary Reference


1. Peng Z, *et al.* The draft genome of the fast-growing non-timber forest species moso bamboo (*Phyllostachys heterocycla*). *Nat. Genet.* **45**, 456-461 (2013).
2. Darling AE, Mau B, Perna NT. ProgressiveMauve: Multiple genome alignment with gene gain, loss and rearrangement. *PLoS One* **5**, e11147 (2010).
3. Wang X, Li C, Zhou C, Li J, Zhang Y. Molecular characterization of the C-glucosylation for puerarin biosynthesis in Pueraria lobata. *Plant J.* **90**, 535-546 (2017).
4. Chen DW, *et al.* Probing the catalytic promiscuity of a regio- and stereospecific *C*-glycosyltransferase from *Mangifera indica*. *Angew. Chem. Int. Edit.* **54**, 12678-12682 (2015).
5. Nagatomo Y, Usui S, Ito T, Kato A, Shimosaka M, Taguchi G. Purification, molecular cloning and functional characterization of flavonoid *C*-glucosyltransferases from *Fagopyrum esculentum* M. (buckwheat) cotyledon. *Plant J.* **80**, 437-448 (2014).
6. Hirade Y, Kotoku N, Terasaka K, Saijo-Hamano Y, Fukumoto A, Mizukami H. Identification and functional analysis of 2-hydroxyflavanone *C*-glucosyltransferase in soybean (*Glycine max*). *FEBS Lett.* **589**, 1778-1786 (2015).
7. Ito T, Fujimoto S, Suito F, Shimosaka M, Taguchi G. *C*-glycosyltransferases catalyzing the formation of di-*C*-glucosyl flavonoids in citrus plants. *Plant J.* **91**, 187-198 (2017).
8. Moheb A, Ibrahim RK, Roy R, Sarhan F. Changes in wheat leaf phenolome in response to cold acclimation. *Phytochemistry* **72**, 2294-2307 (2011).
9. Colombo R, Lancas FM, Yariwake JH. Determination of flavonoids in cultivated sugarcane leaves, bagasse, juice and in transgenic sugarcane by liquid chromatography-UV detection. *J. Chromatogr. A* **1103**, 118-124 (2006).
10. Colombo R, Yariwake JH, Queiroz EF, Ndjoko K, Hostettmann K. On-line identification of further flavone *C*- and *O*-glycosides from sugarcane (*Soccharum officinarum* L., Gramineae) by HPLC-UV-MS. *Phytochem. Analysis* **17**, 337-343 (2006).
11. Ramarathnam N, Osawa T, Namiki M, Kawakishi S. Chemical studies on novel rice hull antioxidants .2. identification of isovitexin, a *C*-glycosyl flavonoid. *J. Agric. Food Chem.* **37**, 316-319 (1989).

12. Kim B, *et al.* Identification and quantification of flavonoids in yellow grain mutant of rice (*Oryza sativa* L.). *Food Chem.* **241**, 154-162 (2018).

13. Harborne JB, Boardley M, Frost S, Holm G. The flavonoids in leaves of diploid *Triticum* species (Gramineae). *Plant System. Evol.***154**, 251-257 (1986).

14. Besson E, *et al. C*-glycosylflavones from *Oryza sativa*. *Phytochemistry* **24**, 1061-1064 (1985).

15. Yang ZG, *et al.* Toward better annotation in plant metabolomics: isolation and structure elucidation of 36 specialized metabolites from *Oryza sativa* (rice) by using MS/MS and NMR analyses. *Metabolomics* **10**, 543-555 (2014).

16. Coutinho ID, Baker JM, Ward JL, Beale MH, Creste S, Cavalheiro AJ. Metabolite profiling of sugarcane genotypes and identification of flavonoid glycosides and phenolic acids. *J. Agric. Food Chem.* **64**, 4198-4206 (2016).

17. Elliger CA, Chan BG, Waiss AC, Lundin RE, Haddon WF. *C*-glycosylflavones from *Zea mays* that inhibit insect development. *Phytochemistry* **19**, 293-297 (1980).

18. Frost S, Harborne JB, King L. Identification of flavonoids in 5 chemical races of cultivated barley. *Hereditas* **85**, 163-168 (1977).

19. Yang Z, Nakabayashi R, Mori T, Takamatsu S, Kitanaka S, Saito K. Metabolome analysis of *Oryza sativa* (rice) using liquid chromatography-mass spectrometry for characterizing organ specificity of flavonoids with anti-inflammatory and anti-oxidant activity. *Chem. Pharm. Bull.* **64**, 952-956 (2016).

20. Waiss AC, *et al.* Maysin, a flavone glycoside from corn silks with antibiotic-activity toward corn earworm (Lepidotera, noctuidae). *J. Econ. Entomol.* **72**, 257-258 (1979).

21. Brazier-Hicks M, Evans KM, Gershater MC, Puschmann H, Steel PG, Edwards R. The *C*-glycosylation of flavonoids in cereals. *J. Biol. Chem.* **284**, 17926-17934 (2009).

22. Ferreyra MLF, Rodriguez E, Casas MI, Labadie G, Grotewold E, Casati P. Identification of a bifunctional maize *C*- and *O*-glucosyltransferase. *J. Biol. Chem.* **288**, 31678-31688 (2013).

23. D'Hont A, *et al.* The banana (*Musa acuminata*) genome and the evolution of monocotyledonous plants. *Nature* **488**, 213-217 (2012).

24. Al-Dous EK, *et al.* De novo genome sequencing and comparative genomics of date palm (*Phoenix dactylifera*). *Nat. Biotech.* **29**, 521-527 (2011).

25. Tamiru M, *et al.* Genome sequencing of the staple food crop white Guinea yam enables the development of a molecular marker for sex determination. *BMC Biol.* **15**, 86 (2017).

26. Zhou W, *et al.* Genome survey sequencing of *Dioscorea zingiberensis*. *Genome* **61**, 567-574 (2018).

27. Cai J, *et al.* The genome sequence of the orchid *Phalaenopsis equestris*. *Nat. Genet.* **47**, 65-72 (2015).

28. Li J, Tian C, Xia Y, Mutanda I, Wang K, Wang Y. Production of plant-specific flavones baicalein and scutellarein in an engineered *E. coli* from available phenylalanine and tyrosine. *Metab. Eng.* **52**, 124-133 (2019).

29. Shih CH, *et al.* Functional characterization of key structural genes in rice flavonoid biosynthesis. *Planta* **228**, 1043-1054 (2008).

30. Lam PY, Liu H, Lo C. Completion of tricin biosynthesis pathway in rice: Cytochrome P450 75B4 is a unique chrysoeriol 5'-hydroxylase. *Plant Physiol.* **168**, 1527-1536 (2015).
