# Supplementary material for: Pathway-specific enzymes from bamboo and crop leaves biosynthesize anti-nociceptive C-glycosylated flavones
Source: Commun Biol. 2020 Mar 6;3:110. doi: 10.1038/s42003-020-0834-3 (PMC7060329; doi:10.1038/s42003-020-0834-3)
Supplement: Supplementary file 3 — Reporting Summary [file 42003_2020_834_MOESM3_ESM.pdf]

nature research

Corresponding author(s): Yong Wang
Last updated by author(s): Jan 24, 2020

Reporting Summary

Nature Research wishes to improve the reproducibility of the work that we publish. This form provides structure for consistency and transparency in reporting. For further information on Nature Research policies, see Authors & Referees and the Editorial Policy Checklist.

Statistics

For all statistical analyses, confirm that the following items are present in the figure legend, table legend, main text, or Methods section.

n/a	Confirmed	
☐	☒	The exact sample size (*n*) for each experimental group/condition, given as a discrete number and unit of measurement
☐	☒	A statement on whether measurements were taken from distinct samples or whether the same sample was measured repeatedly
☒	☐	The statistical test(s) used AND whether they are one- or two-sided<br>*Only common tests should be described solely by name; describe more complex techniques in the Methods section.*
☒	☐	A description of all covariates tested
☐	☒	A description of any assumptions or corrections, such as tests of normality and adjustment for multiple comparisons
☐	☒	A full description of the statistical parameters including central tendency (e.g. means) or other basic estimates (e.g. regression coefficient) AND variation (e.g. standard deviation) or associated estimates of uncertainty (e.g. confidence intervals)
☐	☒	For null hypothesis testing, the test statistic (e.g. *F*, *t*, *r*) with confidence intervals, effect sizes, degrees of freedom and *P* value noted<br>*Give P values as exact values whenever suitable.*
☒	☐	For Bayesian analysis, information on the choice of priors and Markov chain Monte Carlo settings
☒	☐	For hierarchical and complex designs, identification of the appropriate level for tests and full reporting of outcomes
☒	☐	Estimates of effect sizes (e.g. Cohen's *d*, Pearson's *r*), indicating how they were calculated

Our web collection on statistics for biologists contains articles on many of the points above.

Software and code

Policy information about availability of computer code

Data collection	No software was used
Data analysis	MCScanx; Mega X; GraphPad Prism 7

For manuscripts utilizing custom algorithms or software that are central to the research but not yet described in published literature, software must be made available to editors/reviewers. We strongly encourage code deposition in a community repository (e.g. GitHub). See the Nature Research guidelines for submitting code & software for further information.

Data

Policy information about availability of data

All manuscripts must include a data availability statement. This statement should provide the following information, where applicable:

- Accession codes, unique identifiers, or web links for publicly available datasets
- A list of figures that have associated raw data
- A description of any restrictions on data availability

The gene sequences of Phyllostachys bamboos were deposited in GenBank under the following accession numbers: PhCGT1, MK616588; PhCGT2, MK616589; MK616593; PhF2H, MK628906; PhF3'H, MK636710; PhCGT3, MK616590; PhCGT4, MK616591; PgCGT1, MK616592; PpCGT1,

Field-specific reporting

Please select the one below that is the best fit for your research. If you are not sure, read the appropriate sections before making your selection.

☒ Life sciences ☐ Behavioural & social sciences ☐ Ecological, evolutionary & environmental sciences

Life sciences study design

All studies must disclose on these points even when the disclosure is negative.

Sample size	Samples size were determined according to the needs of the experiment itself and the relevant references. Unpaired t test was used.
Data exclusions	No data were excluded.
Replication	The experiment was repeated three times and similar results were obtained.
Randomization	Samples were allocated into experimental groups randomly.
Blinding	The investigators were blinded to group allocation during data collection and analysis.

Reporting for specific materials, systems and methods

We require information from authors about some types of materials, experimental systems and methods used in many studies. Here, indicate whether each material, system or method listed is relevant to your study. If you are not sure if a list item applies to your research, read the appropriate section before selecting a response.

Materials & experimental systems

n/a	Involved in the study	
☐	☒	Antibodies
☒	☐	Eukaryotic cell lines
☒	☐	Palaeontology
☐	☒	Animals and other organisms
☒	☐	Human research participants
☒	☐	Clinical data

Methods

n/a	Involved in the study	
☒	☐	ChIP-seq
☒	☐	Flow cytometry
☒	☐	MRI-based neuroimaging

Antibodies

Antibodies used

GluN2B:CST,4212,3; TRVP1:Abcam,ab6166,GR3229328-1;GAPDH:proteintech,60004-1-Ig,1E6D9;Beyotime Biotechnology,A0208; Beyotime Biotechnology,A0216.

Validation

NMDA Receptor 2B (GluN2B) (D15B3) Rabbit mAb 4212: NMDA Receptor2B (GluN2B) (D15B3) Rabbit mAb detects endogenous levels of total NMDAR2B protein. Monoclonal antibody is produced by immunizing animals with a synthetic peptide corresponding to residues surrounding Leu1134 of human NMDAR2B protein.
Relevant citations:
(1) Liu, X.B. et al. (2004) J. Neurosci. 24, 8885–8895.
(2) Westphal, R.S. et al. (1999) Science 285, 93–96.
(3) Tingley, W.G. et al. (1997) J. Biol. Chem. 272, 5157–5166.
(4) Hisatsune, C. et al. (1997) J. Biol. Chem. 272, 20805–20810.
(5) Raman, I.M. et al. (1996) Neuron 16, 415–421.
(6) Makhinson, M. et al. (1999) J. Neurosci. 19, 2500–2510.
(7) Takasu, M.A. et al. (2002) Science 295, 491–495;
Anti-VR1 antibody ab6166: Widely expressed at low levels. Expression is elevated in dorsal root ganglia. In skin, expressed in cutaneous sensory nerve fibers, mast cells, epidermal keratinocytes, dermal blood vessels, the inner root sheet and the infundibulum of hair follicles, differentiated sebocytes, sweat gland ducts, and the secretory portion of eccrine sweat glands (at protein level).
Relevant citations:
[1]Dos Anjos-Garcia T & Coimbra NC Opposing roles of dorsomedial hypothalamic CB1 and TRPV1 receptors in anandamide signaling during the panic-like response elicited in mice by Brazilian rainbow Boidae snakes. Psychopharmacology (Berl) N/A:N/A (2019). Read more (PubMed: 30694375)
[2]Zhong B et al. TRPV1 protects renal ischemia-reperfusion injury in diet-induced obese mice by enhancing CGRP release and increasing renal blood flow. PeerJ 7:e6505 (2019). Read more (PubMed: 30834186)
GAPDH Monoclonal ANTIBODY:Tested Applications: FC, IF, IHC, IP, WB, ELISA Cited Applications: IF, IHC, IP, WB Species Specificity: human, mouse, rat, zebrafish, yeast,plant Cited Species: beagle, carp, chicken, cow, Cynomorium songaricum Rupr, Cyprinus carpio, deer, dog, Eelworm, Goat
Relevant citations:
[1] Tian Tian , J Cell Biochem.
[2] Jaehwan You , J Virol.
[3] Junhua Guo, Mol Cells .

Animals and other organisms

Policy information about studies involving animals; ARRIVE guidelines recommended for reporting animal research

Laboratory animals	6-8 weeks old ICR male mice
Wild animals	This study did not involve wild animals
Field-collected samples	P. meyeri was collected from Anji County (E 119.64°, N 30.50°), Zhejiang Province, China in Oct. 2017 and March, 2018. P. glauca, P. edulis (Synonym: P. heterocycla cv. Pubescens), P. prominens and P. bissetii were collected from Shanghai Chenshan Botanical Gardens in April. 2018.
Ethics oversight	Shanghai Ocean University

Note that full information on the approval of the study protocol must also be provided in the manuscript.
